# Match-play data according to playing categories in badminton: a systematic review

**DOI:** 10.3389/fspor.2025.1466778

**Published:** 2025-02-26

**Authors:** Bagus Winata, Joana Brochhagen, Tommy Apriantono, Matthias Wilhelm Hoppe

**Affiliations:** ^1^ Department of Sports Science, School of Pharmacy, Institut Teknologi Bandung, Bandung, Indonesia; ^2^ Department of Exercise Science, Institute of Sport Science and Motology, Philipps University of Marburg, Marburg, Germany; ^3^ Movement and Training Science, Faculty of Sport Science, Leipzig University, Leipzig, Germany

**Keywords:** game characteristics, metabolism, notational analysis, physical demands, physiology, racquet sports

## Abstract

**Introduction:**

This systematic review aimed to investigate differences in match-play data according to the five playing categories in badminton.

**Materials and methods:**

The systematic review was conducted following the Preferred Reporting Items for Systematic Reviews and Meta-Analyses (PRISMA) guidelines. Searches were conducted on ScienceDirect, PubMed, Google Scholar, Scopus, Web of Science, and Cochrane Library databases. Studies assessing technical-tactical actions, activity profiles, or external and internal loads as match-play outcome measures according to the five playing categories in badminton were deemed eligible. Quality assessment was performed using a modified version of the AMSTAR-2 checklist to compare the outcome measures, effect sizes (ES) and associated 95% confidence intervals were calculated.

**Results:**

Of the 12,967 studies that were identified, 34 met the eligibility criteria. Among these, 29 and five were rated as excellent and good quality, respectively. Some individual ESs of activity profiles showed up to large differences (ES ≤ 4.52) favouring the men's compared with the women's singles category. Some individual ESs of activity profiles showed up to large differences (ES ≤ -2.72) favouring the women's doubles category compared with other doubles categories. The overall ESs for the activity profiles were large (ES = −0.76 to −0.90), favouring the doubles over the singles categories in both sexes.

**Discussion:**

There are up to large differences in match-play data according to the five playing categories in badminton, each category placing specific demands on the players. Thus, each category requires specific training and testing procedures, what should be considered by scientists and coaches.

## 1 Introduction

Since 1992, badminton has been part of the Olympic Games and has developed into a racquet sport with a professional structure and high level of competition (1). It has been estimated that over 200 million people play this sport recreationally and more than 7,000 athletes compete in hundreds of international and national competitions each year (1). To compete, the intermittent characteristics of badminton require optimised physical, technical-tactical, and psychological factors (2–4). An important aspect of badminton is the five different playing categories: men's and women's singles and men's, women's, and mixed doubles (5). Without strong evidence, the different playing categories are assumed to place specific demands on the players (1). Thereon, specific training and testing procedures are required to prepare the players (6, 7). Generally, such procedures should mimic particular playing demands (8), thereby requiring knowledge of match-play data (9, 10).

Badminton match-play data can be categorised into four groups: (1) technical-tactical actions, (2) activity profiles, (3) external (mechanical), and (4) internal (physiological) loads (9, 11). The four groups are interrelated (12). For example, technical-tactical actions such as smash, drive, drop, lob, and clear shots affect the activity profiles regarding the match duration, rally time, and work density (13–15). Furthermore, the resulting activity profiles affect the external and internal loads that players must meet (1, 9, 10). Finally, internal loads can induce long-term adaptations that can influence all the aforementioned aspects (10). Thus, a comprehensive understanding of these variable groups and their interactions is valuable to consider for scientists and coaches in badminton, when aiming to design specific training and testing procedures for the playing categories and other important influencing attributes of the players such as sex, playing level, and age (1, 9, 16–18). As evidence, analyzing match-play data helps identify the specific demands placed on players, enabling them to enhance performance through physiological adaptations and reduce the risk of injuries (17). Unfortunately, evidence on match-play data in badminton is limited; especially, regarding the specific demands of the five playing categories (1). Thus, more evidence-based research is needed.

To our knowledge, there are three reviews on badminton match-play. One systematic review investigated the effects of badminton on health outcomes and discovered that this sport improves cardiopulmonary function and physical abilities such as endurance and strength (19). A further review on general playing characterises of badminton was narrative in its nature (1) and the other solely focused on internal loads across several racquet sports (20). While previous reviews have provided evidence of the health benefits (19), general characteristics (1), and internal loads of badminton (20), there is no systematic overview regarding match-play data in badminton yet. Consequently, how the existing literature on badminton match-play data differs especially based on the five playing categories is still unclear. This problem creates ambiguity and debates among scientists and coaches (1). Therefore, a systematic literature review of match-play data concerning the five playing categories in badminton is warranted.

Thus, this systematic review aimed to investigate differences in match-play data according to the five playing categories in badminton.

## 2 Materials and methods

### 2.1 Research design and search strategy

The systematic review was conducted following the Preferred Reporting Items for Systematic Reviews and Meta-Analyses (PRISMA) guidelines (21). The initial literature search was conducted in databases, including ScienceDirect, PubMed, Google Scholar, Scopus, Web of Science, and Cochrane Library on 1 June 2023. Additionally, to ensure that all actual available literature was included, an update was conducted immediately before submission on July 2024. The P = Population, I = Intervention, C = Comparisons, and O = Outcomes (PICO) scheme (21) was used to develop the search lines. Search terms were created by linking category sections with the Boolean operator “AND” to ensure that at least one term from each section appeared in searches, while the “OR” operator was used to link terms within a section (Table 1). The received entries were downloaded to a citation manager (Clarivate Analytics, Endnote X9, London, UK), and duplicates were removed. Furthermore, the “related citations” feature of PubMed was used to identify further relevant studies. A spreadsheet (Microsoft Office, Excel 2016, Redmond, WA, USA) was created to manage the detected studies following the developed PICO scheme. The titles, abstracts, and full texts of the selected studies were screened based on the defined eligibility criteria and studies considered unsuitable were excluded. Additionally, the reference lists of eligible studies were reviewed to identify relevant studies that were not detected by the search line. Any studies using the pre-2006 scoring system were excluded due to significant differences in playing time, which could affect the match-play outcome measures (22). All data were independently extracted by two authors (BW and TA). In terms of disagreements, a third author (MWH) was added and it was discussed until a consensus was reached. This proceed was also applied to the study quality assessment described below.

**Table 1:** Search line according to the PICO scheme.

PICO scheme		Category	Keyword search
P	Badminton players	Sex	“Males” OR “men” OR “females” OR “women”
		Playing level	“Recreational” OR “hobby” OR “amateur” OR “professional” OR “elite”
		Age	“Junior” OR “adolescent” OR “adult” OR “senior”
I	Match-play	Playing context	“Official” OR “competitive” OR “simulated” OR “training”
C	Participants groups	Badminton playing categories	“Singles” OR “doubles” OR “mixed”
O	Match-play outcome measures	Technical-tactical actions	“Smash” OR “drive” OR “drop” OR “lob” OR “clear” OR “net” OR “forced error” OR “unforced error” OR “shuttlecock placement”
		Activity profiles	“Match duration” OR “number of rallies” OR “rally time” OR “shot” OR “stroke” OR “shot per rally” OR “stroke per rally” OR “shot frequency” OR “rest time” OR “work to rest ratio” OR “effective playing” OR “work density”
		External loads	“Jump” OR “lunges” OR “speed” OR “velocity” OR “acceleration” OR “deceleration” OR “distance covered”
		Internal loads	“Oxygen uptake” OR “VO_2_″ OR “tidal volume” OR “V_T_” OR “respiratory frequency” OR “f_B_” OR “minute ventilation” OR “V˙_E_” OR “carbon dioxide production” OR “V˙CO_2_” OR “respiratory exchange ratio” OR “RER” OR “respiratory ratio” OR “RQ” OR “ventilatory equivalents for oxygen” OR “V˙E/V˙O_2_” OR “ventilatory equivalents for carbon dioxide” OR “V˙E/V˙CO_2_” OR “oxygen end-tidal pressure” OR “PetO_2_” OR “carbon dioxide end-tidal pressure” OR “PetCO_2_” OR “heart rate” OR “blood pressure” OR “blood lactate concentration” OR “ratings of perceived exertion” OR “RPE” OR “energy expenditure” OR “energy supply” OR “aerobic” OR “oxidative” OR “anaerobic” OR “lactic” OR “alactic” OR “creatine phosphate” OR “phosphocreatine system” OR “PCr” Or “anaerobic glycolysis” OR “blood hormone” OR “testosterone” OR “estrogen” OR “cortisol” OR “thyroid levels” OR “fluid electrolyte balance” OR “sodium” OR “calcium” OR “potassium” OR “chloride” OR “phosphate” OR “magnesium” OR “body composition” OR “body height” OR “body weight” OR “body mass index” OR “BMI” OR “body mass” OR “lean mass” OR “fat-mass” OR “fat-free Mass”

### 2.2 Eligibility criteria

This review included cross-sectional and longitudinal studies involving both sexes, playing level, all ages of badminton players investigated during match-play. The specific eligibility criteria included studies: (1) written in English; (2) with ethical approval (except for retrospective studies); (3) involving non-injured or non-paralympic players; (4) including the five badminton playing categories; (5) investigating official or simulated matches without experimental approaches; and (6) involving technical-tactical actions, activity profiles, or external and internal loads as match-play outcome measures.

### 2.3 Study quality assessment

A modified version of the AMSTAR-2 checklist (23) was used to assess the study quality based on 16 specific questions related to: (1) clarity of purpose; (2) relevance of background literature; (3) appropriate study design; (4) study sample; (5) sample size justification; (6) informed consent procedure (if any); (7) reliability and (8) validity of outcome measures; (9) detailed method description; (10) results reporting; (11) analysis methods; (12) description of practical importance; (13) description of drop-outs (if any); (14) appropriately drawn conclusions; (15) implications for practice; and (16) acknowledgement of study limitations. Previous study was modified critical review components into a single score, which proved effective for assessing the risk of bias in observational studies (10). Each question was scored using binary values (0 = no, 1 = yes), except for questions 6 and 13, for which “not applicable” was also an option. Finally, the results were converted into a percentage score by summing all individual scores and dividing by the maximum possible score; thus, a higher percentage indicated a higher study quality (10). As previously conducted, the scores were divided into three methodological quality categories: low (≤50%), good (51% to 75%), and excellent (>75%) (10, 24).

### 2.4 Data extraction

The data were extracted using the PICO scheme. The following data were collected: P = sample size, sex, playing level, and age; I = playing context (official or simulated match-play); C = single, double, or mixed playing categories; and O = main outcome measure regarding technical-tactical actions, activity profiles, and external and internal loads. If published data were unclear or missing, the corresponding authors were contacted via e-mail.

### 2.5 Data synthesis

The outcome measures were categorised into the five playing categories in badminton. Additionally, sex (men and women) and playing level were considered, whereby the latter was clustered into world-class, elite/international level, highly trained/national level, trained/developmental, and recreationally active players, as previously recommended (25).

### 2.6 Statistical analysis

Since a meta-analysis could not be conducted due to the large heterogeneity of the included studies and their data, effect sizes (ES) and associated 95% confidence intervals (CI) were alternatively calculated to compare differences in means (26), as conducted in previous systematic reviews with similar applied sport science purposes before (27, 28). The main advantage of that alternative statistical approach is that ESs can easily be computed from the means and standard deviations of the included original studies, which enhances the transparency and reliability of the outcome statistics of reviews. With respect to the computation, both individual and overall ESs were computed. Therefore, the mean differences were divided by the average standard deviations (29), with pooled baseline standard deviations (30). Based on established criteria (30), ESs were interpreted as small (<0.40), moderate (0.40–0.70), and large (>0.70). For validity, ESs were calculated only for means based on, at least in part, two studies or comparisons. Microsoft Excel 2016 (Microsoft, Redmond, WA, USA) was used for all the calculations.

## 3 Results

### 3.1 Literature search

**Figure 1:**
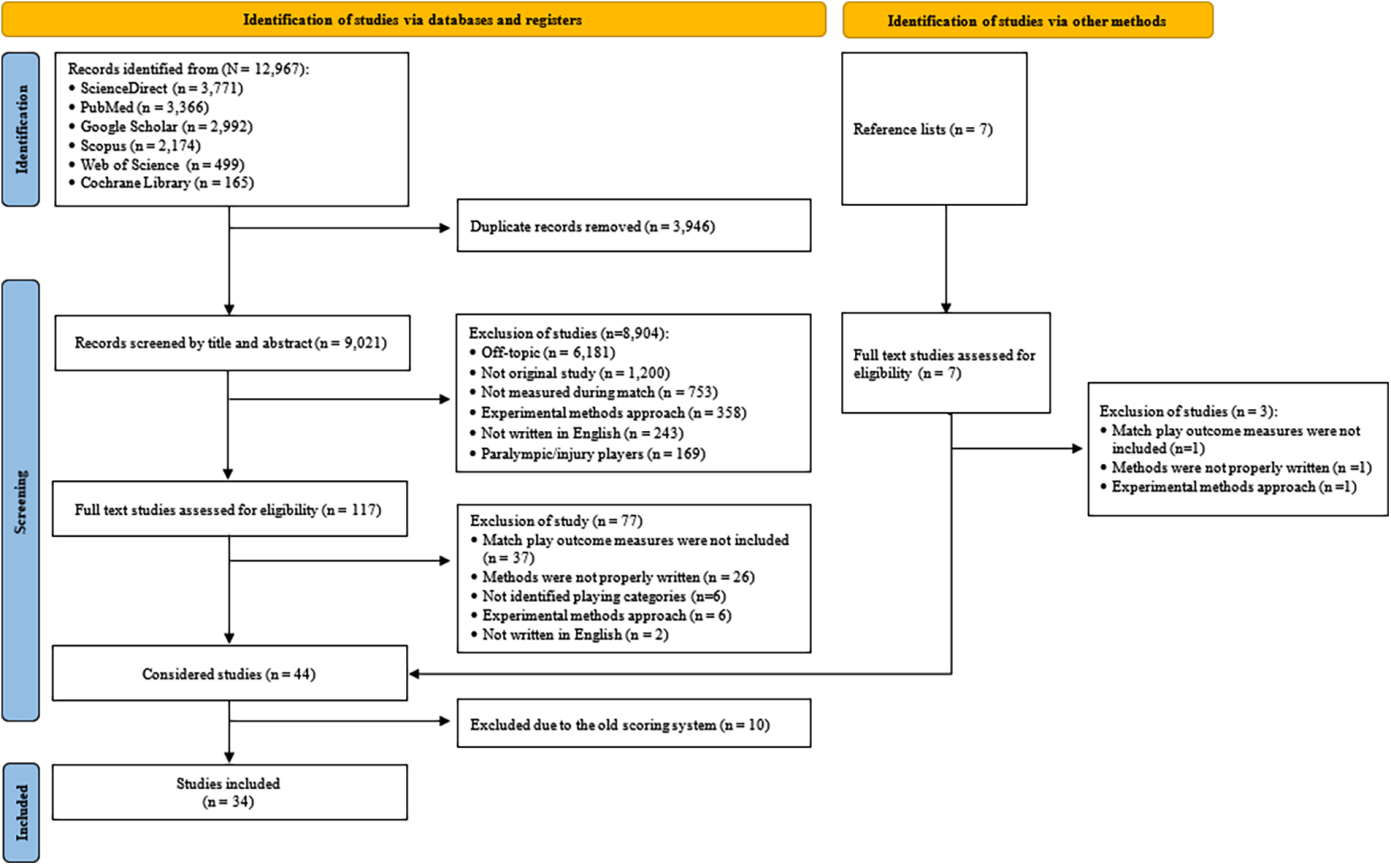
Flow chart of the literature search strategy adopted from the PRISMA-guidelines.

Figure 1 illustrates the results of the literature search. Initially, 12,967 studies were identified, with 3,946 removed because of duplication, leaving 9,021 studies. These studies were screened by title and abstract against the defined eligibility criteria, resulting in the exclusion of 8,904 studies. The remaining 117 studies underwent full-text screening, with 77 excluded based on the exclusion criteria. From the 40 studies considered, seven additional studies were obtained from the reference lists. Of these, three were excluded for not meeting the eligibility criteria, resulting in 44 studies. However, 10 of these 44 studies were removed because they used an outdated badminton scoring system that existed until 2006 (13–15, 22, 31–36). Finally, the remaining 34 studies were considered for the quality assessment (2–5, 37–66).

### 3.2 Study quality of the studies

Table 2 presents the results of the quality assessment of the 34 included studies. The mean quality score was 83.1%. No study had a score of 100%, but 29 studies (2–5, 37–45, 47, 50–62, 65, 66) were rated as excellent quality. Five studies (46, 48, 49, 63, 64) were of good quality; no study was of low quality. Studies with higher scores had practical implications (Question 15) and limitations (Question 16). No study provided drop-out rates, making question 13 always “not applicable”. Fifteen studies (2, 4, 42, 43, 46, 48, 49, 51, 52, 54, 58, 62, 64–66) showed “not applicable” for question 6 owing to their retrospective designs. All studies lost quality points on sample size justification (Question 5), and 12 studies (2, 4, 43, 46, 48, 49, 52, 54, 58, 62, 64, 65) lost points owing to sample description (Question 4).

**Table 2:** Study quality assessment using the AMSTAR-2 checklist for the 34 included studies.

Study	Question																Score (%)	Quality
	1	2	3	4	5	6	7	8	9	10	11	12	13	14	15	16		
Abdullahi et al. (3)	Y	Y	Y	Y	N	Y	Y	Y	Y	Y	Y	Y	N/A	Y	Y	Y	93.3	Excellent
Abdullahi et al. (5)	Y	Y	Y	Y	N	Y	Y	Y	Y	Y	Y	Y	N/A	Y	Y	Y	93.3	Excellent
Abián et al. (42)	Y	Y	Y	Y	N	N/A	Y	Y	Y	Y	Y	Y	N/A	Y	Y	N	85.7	Excellent
Abian-Vicen et al. (2)	Y	Y	Y	N	N	N/A	Y	Y	Y	Y	Y	Y	N/A	Y	Y	N	78.6	Excellent
Abian-Vicen et al. (40)	Y	Y	Y	Y	N	Y	Y	Y	Y	Y	Y	Y	N/A	Y	Y	N	86.7	Excellent
Abián-Vicén et al. (38)	Y	Y	Y	Y	N	Y	Y	Y	Y	Y	Y	Y	N/A	Y	N	N	80.0	Excellent
Abián-Vicén et al. (4)	Y	Y	Y	N	N	N/A	Y	Y	Y	Y	Y	Y	N/A	Y	Y	N	78.6	Excellent
Apriantono et al. (53)	Y	Y	Y	Y	N	N	Y	Y	Y	N	Y	Y	N/A	Y	Y	Y	80.0	Excellent
Bisschoff et al. (45)	Y	Y	Y	Y	N	N	Y	Y	Y	Y	Y	Y	N/A	Y	Y	Y	86.7	Excellent
Bisschoff et al. (44)	Y	Y	Y	Y	N	N	Y	Y	Y	Y	Y	Y	N/A	Y	Y	Y	86.7	Excellent
Chiminazzo et al. (49)	Y	Y	Y	N	N	N/A	Y	Y	Y	Y	Y	Y	N/A	Y	N	N	71.4	Good
Deka et al. (47)	Y	Y	Y	Y	N	N	Y	Y	Y	Y	Y	Y	N/A	Y	N	Y	80.0	Excellent
Faude et al. (37)	Y	Y	Y	Y	N	Y	Y	Y	Y	Y	Y	Y	N/A	Y	N	N	80.0	Excellent
Fernandez-Fernandez et al. (41)	Y	Y	Y	Y	N	Y	Y	Y	Y	Y	Y	Y	N/A	Y	Y	N	86.7	Excellent
Fu et al. (57)	Y	Y	Y	Y	N	Y	Y	Y	Y	Y	Y	Y	N/A	Y	Y	Y	93.3	Excellent
Gawin et al. (43)	Y	Y	Y	N	N	N/A	Y	Y	Y	Y	Y	Y	N/A	Y	Y	N	78.6	Excellent
Gomez et al. (58)	Y	Y	Y	N	N	N/A	Y	Y	Y	Y	Y	Y	N/A	Y	Y	Y	85.7	Excellent
Gómez-Ruano et al. (54)	Y	Y	Y	N	N	N/A	Y	Y	Y	Y	Y	Y	N/A	Y	Y	Y	85.7	Excellent
Green et al. (60)	Y	Y	Y	Y	N	Y	Y	Y	Y	Y	Y	Y	N/A	Y	Y	Y	93.3	Excellent
Hoffmann et al. (65)	Y	Y	Y	N	N	N/A	Y	Y	Y	Y	Y	Y	N/A	Y	Y	Y	85.7	Excellent
Jiménez et al. (39)	Y	Y	Y	Y	N	Y	Y	Y	Y	Y	Y	Y	N/A	Y	N	N	80.0	Excellent
Kui et al. (63)	Y	Y	Y	Y	N	Y	Y	N	Y	N	N	Y	N/A	Y	Y	Y	73.3	Good
Le Mansec et al. (62)	Y	Y	Y	N	N	N/A	Y	Y	Y	Y	Y	Y	N/A	Y	Y	Y	85.7	Excellent
Leong et al. (46)	Y	Y	Y	N	N	N/A	Y	Y	N	Y	Y	Y	N/A	Y	Y	N	71.4	Good
Lin et al. (61)	Y	Y	Y	Y	N	Y	Y	Y	Y	Y	Y	Y	N/A	Y	N	Y	86.7	Excellent
Nagano et al. (55)	Y	Y	Y	Y	N	Y	Y	Y	Y	Y	Y	Y	N/A	Y	Y	Y	93.3	Excellent
Phomsoupha et al. (50)	Y	Y	Y	Y	N	Y	Y	Y	Y	Y	Y	Y	N/A	Y	N	N	80.0	Excellent
Rojas-Valverde et al. (56)	Y	Y	Y	Y	N	Y	Y	Y	Y	Y	Y	Y	N/A	Y	Y	Y	93.3	Excellent
Sales et al. (59)	Y	Y	Y	Y	N	Y	Y	Y	Y	Y	Y	Y	N/A	Y	Y	Y	93.3	Excellent
Torres-Luque et al. (51)	Y	Y	Y	Y	N	N/A	Y	Y	Y	Y	Y	Y	N/A	Y	Y	N	85.7	Excellent
Torres-Luque et al. (52)	Y	Y	Y	N	N	N/A	Y	Y	Y	Y	Y	Y	N/A	Y	Y	N	78.6	Excellent
Valldecabres et al. (48)	Y	N	Y	N	N	N/A	Y	Y	N	N	N	Y	N/A	Y	Y	Y	57.1	Good
Xiang-Qian Xu et al. (64)	Y	Y	Y	N	N	N/A	Y	Y	N	Y	Y	Y	N/A	Y	Y	N	71.4	Good
Zhang et al. (66)	Y	Y	Y	Y	N	N/A	Y	Y	Y	Y	Y	Y	N/A	Y	Y	N	85.7	Excellent

Y, yes; N, no; N/A, not applicable.

### 3.3 Study characteristics

Tables 3–5 summarise the characteristics of the 34 studies using the PICO scheme. Fourteen studies (4, 42, 43, 46, 48, 49, 51, 52, 54, 58, 62, 64–66) did not provide data regarding sample size. The remaining 20 studies reported data on 362 players, including 244 men and 118 women. The mean age of all players was 20.6 ± 4.9 years, with 21.5 ± 5.4 and 18.9 ± 3.5 years for men and women, respectively. Sixteen studies (2, 4, 37–41, 43, 48, 51, 52, 56, 57, 59, 62, 63) investigated men and women; 14 studies (3, 5, 42, 44–47, 49, 50, 53, 58, 60, 61, 65) investigated only men; and four studies (54, 55, 64, 66) reported data on only women. Regarding playing level, world-class players were most frequently observed in 14 studies (2, 4, 40, 42–45, 49–52, 54, 58, 64); twelve studies (3, 5, 37–39, 46, 48, 53, 61, 62, 65, 66) focused on elite/international players; seven (41, 55–57, 59, 60, 63) on highly trained/national players; and one study (47) investigated recreationally active players. No study investigated players at the training or developmental levels.

**Table 3:** Study characteristics of the 34 included studies concerning the population, intervention, and comparison.

Authors	Population (sample size, sex, playing level, age)	Intervention (official or simulated match-play)	Comparison (singles, doubles, or mixed playing category)
Abdullahi et al. (3)	12 men elite/international level African badminton players (24.4 ± 4.6 years)	Five official badminton matches of the All-Africa Senior Badminton Championships 2014	Comparison among single playing categories regarding descriptive tactical actions and activity profiles data.
Abdullahi et al. (5)	21 men elite/international level African badminton players (23.2 ± 3.6 years)	46 official badminton matches	Comparison among single playing categories regarding descriptive of internal and external loads.
Abián et al. (42)	Men world class badminton players of unknown age and sample size	40 official badminton matches (20 matches in the 2008 Beijing Olympic Games, 20 matches in the 2012 London Olympic Games)	Comparison among single playing categories regarding activity profiles and technical-tactical actions in the two Olympic games.
Abian-Vicen et al. (2)	10 men and 10 women world class badminton players of unknown age	20 official badminton matches of the 2008 Beijing Olympic Games	Comparison among single playing categories regarding the differences between the activity profiles and technical-tactical actions in the games of each match and both playing categories.
Abian-Vicen et al. (40)	13 world class Spanish badminton players (6 men, 7 women; 23.0 ± 4.8 years)	Official Spanish Badminton Championship of the second round and quarterfinal match	Comparison among single playing categories regarding the relationship of internal loads between the first factor (pre-match vs. post-match) and the second factor (second round vs. quarterfinals).
Abián-Vicén et al. (38)	46 men highly trained/national level Spanish badminton players (22.7 ± 4.2 years), 24 women highly trained/national level Spanish badminton players (23.0 ± 5.7 years)	Official Spanish Badminton Championships	Comparison among single playing categories regarding the relationship of internal loads between the first (pre-match vs. post-match) and second factors (men's singles vs. women's singles).
Abián-Vicén et al. (4)	Men and women world class badminton players of unknown age and sample size	48 official badminton matches from the quarterfinals to the finals of the 2008 Beijing, 2012 London, and 2016 Rio Olympic Games	Comparison among double playing categories regarding the differences between the activity profiles variables in the two playing categories and among the three Olympics Games.
Apriantono et al. (53)	12 men elite/international level Indonesian badminton players (16.5 ± 0.6 years)	Simulated badminton match	Comparison among double playing categories regarding the differences of technical-tactical actions and internal loads.
Bisschoff et al. (45)	22 men world class African badminton players (23.3 ± 3.9 years)	46 official badminton matches	Comparison among single playing categories regarding the differences in internal loads between successful players and less successful players.
Bisschoff et al. (44)	22 men world class African badminton players (23.3 ± 3.9 years)	46 official badminton matches	Comparison among single playing categories regarding differences of internal loads in pre-match, in-match, resting, and post-match.
Chiminazzo et al. (49)	Men world class badminton players of unknown age and sample size	56 official video badminton matches in the 2016 Rio Olympic Games (43 video matches in the group stage, 13 video matches in the play-off phase)	Comparison among single playing categories regarding differences of technical-tactical actions and activity profiles in groups and play-offs.
Deka et al. (47)	14 men recreationally active badminton players (35.9 ± 6.6 years)	30 min simulated badminton match	Comparison among single playing categories regarding differences in internal and external loads data in the first, middle, and last 10 min, 15 min, and 30 min of the match.
Faude et al. (37)	4 men elite/international level badminton players (21.3 ± 1.7 years), 8 women elite/international level badminton players (21.8 ± 2.1 years)	Simulated badminton match	Comparison among single playing categories regarding differences in internal loads between men and women players, as well as between players who won and lost matches.
Fernandez-Fernandez et al. (41)	8 men highly trained/national level badminton players (16.0 ± 1.4 years), 8 women highly trained/national level badminton players (16.0 ± 2.3 years)	Simulated badminton match	Comparison among single playing categories regarding differences in activity profiles and internal loads between men and women players.
Fu et al. (57)	8 men highly trained/national level badminton players (18.2 ± 3.4 years), 6 women highly trained/national level badminton players (16.5 ± 2.5 years)	Simulated badminton match	Comparison among single playing categories regarding differences in external and internal loads between men and women players, as well as between players who won and lost matches.
Gawin et al. (43)	Men and women world class badminton players of unknown age and sample size	50 official videos badminton matches from 2010 to 2012 in the world-series tournaments (10 videos for each badminton playing category)	Comparison among five badminton playing categories regarding differences of activity profiles variable among each playing category.
Gómez et al. (58)	Men world class badminton players of unknown age and sample size	14 official video badminton matches in the 2016 Rio Olympics Games	Comparison among single playing categories regarding differences of tournament stage, game number, and game half on technical-tactical actions, and activity profile variables.
Gómez-Ruano et al. (54)	Women world class badminton players of unknown age and sample size	14 official video badminton matches of the 2016 Rio Olympic Games matches from the group stage, quarterfinal, semi-finals, and final matches phases who played by the three medallists (gold, silver, and bronze)	Comparison among single playing categories regarding the relationship between the technical-tactical actions (serving player) and activity profiles.
Green et al. (60)	10 men highly trained/national level British badminton players (14.0 ± 1.2 years)	Simulated badminton match	Comparison among single playing categories regarding differences of internal loads and activity profile variables in the first and second games of the simulated match-play and for the total game.
Hoffmann et al. (65)	Men elite/international level badminton players of unknown age and sample size	56 official videos badminton matches from 2006 to 2017 World Championships	Comparison among single playing categories regarding the relationship between the year, place where the matches were played and activity profiles.
Jiménez et al. (39)	27 men elite/international level badminton players (24.5 ± 4.0 years), 23 women elite/international level badminton players (23.6 ± 3.7 years)	Official badminton match	Comparison among single playing categories regarding the relationship between testosterone and cortisol levels in both sexes and match results (victory and defeat).
Kui et al. (63)	4 highly trained/national level badminton players (2 men, 2 women; 17.5 years)	Simulated badminton match	Comparison among single playing categories regarding descriptive data of technical-tactical actions, activity profiles, and internal loads.
Le Mansec et al. (62)	Men and women elite/international level badminton players of unknown age and sample size	Seven official videos badminton matches from the 2016 European Badminton Championship	Comparison among five playing categories regarding differences of the activity profiles, and comparison among doubles playing categories regarding differences of the technical-tactical actions and activity profiles.
Leong et al. (46)	Men elite/international level and highly trained/national level of unknown age and sample size	14 official badminton matches	Comparison among single playing categories regarding differences of activity profiles between professional and junior players.
Lin et al. (61)	10 men elite/international level Australian badminton players (26.4 ± 5.3 years)	40 simulated badminton matches	Comparison among single playing categories regarding differences of external and internal loads data between before and after the matches.
Nagano et al. (55)	10 women highly trained/national level badminton players (15.8 ± 1.0 years)	Simulated badminton match	Comparison among single playing categories regarding the descriptive of external loads data.
Phomsoupha et al. (50)	12 men world class badminton players (25.3 ± 3.2 years)	Simulated badminton match	Comparison among single playing categories regarding differences of technical-tactical actions, activity profiles, and internal loads in each session (10 min, 20 min, 30 min, 40 min, 50 min, and 60 min of badminton match).
Rojas-Valverde et al. (56)	24 highly trained/national level Spanish badminton players (10 men, 14 women; 16.2 ± 0.8 years)	Official tournament of Badminton World Federation IBERDROLA Spanish 2018	Comparison among single playing categories regarding the relationship of external and internal loads data in the match games and sex-related.
Sales et al. (59)	6 men highly trained/national level Brazilian badminton players (14.4 ± 2.1 years), 6 women highly trained/national level Brazilian badminton players (15.7 ± 1.7 years)	Simulated and official badminton matches	Comparison among single playing categories regarding the internal load data in official matches and the training sessions (multi-shuttlecock, technical-tactical actions, and physical training).
Torres-Luque et al. (51)	Men and women world class badminton players of unknown age and sample size	Official badminton matches in the Rio 2016 Olympic Games	Comparison among five playing categories regarding different match activity profiles in group phase vs. eliminatory phase.
Torres-Luque et al. (52)	Men and women world class badminton players of unknown age and sample size	Official badminton matches in the London 2012 and the Rio 2016 Olympic Games	Comparison among single playing categories regarding the differences of activity profiles between sexes in Olympic Games.
Valldecabres et al. (48)	Men and women world class badminton players of unknown age and sample size	Official badminton matches in the 2015 Badminton World Championship	Comparison among single playing categories regarding the differences of technical-tactical actions, and activity profiles between sexes.
Xiang-Qian Xu et al. (64)	Women world class badminton players of unknown age and sample size	58 official videos badminton matches in the Tokyo 2020 Olympic Games	Comparison among single playing categories regarding different match activity profiles in groups phase vs. eliminatory phase.
Zhang et al. (66)	Women elite/international level badminton players of unknown age and sample size	40 official videos badminton matches from 2018 to 2021 Badminton World Federation World Tour	Comparison among single playing categories regarding different technical-tactical actions in two different singles game formats (two right-handers and opposite handedness).

Min, minutes.

**Table 4:** Study characteristics of the 34 included studies concerning the match-play outcome measures of singles category in both sexes.

Authors	Outcome (technical-tactical actions)	Outcome (activity profiles)	Outcome (external loads)	Outcome (internal loads)
Men's singles category				
Abián et al. (42)	The differences between any of the shots (smash, clear, drop, net, drive, lob, and error shots) between the Beijing and London Olympics Games were not significant; the lob shot was more common in London (Beijing: 2.31 ± 1.74%, London: 3.92 ± 4.31%, *p* = 0.06) and net shots were more common in Beijing (Beijing: 16.03 ± 6.6%, London: 13.32 ± 5.38%, *p* = 0.08); unforced errors (Beijing: 41.01 ± 9.46%, London: 42.64 ± 8.89%, *p* = 0.548) and the smash (Beijing: 29.09 ± 8.43%, London: 27.84 ± 8.14%, *p* = 0.317) were the most common last shots of a rally	The London Olympic Games had significantly higher values of the following variables than the Beijing Olympics Games: match duration (1,260.3 ± 267.1 vs. 1,124.6 ± 229.9 s, *p* < 0.05), real-time played (354.7 ± 87.5 vs. 306.9 ± 45.7 s, *p* < 0.05), rally time (10.4 ± 2.1 vs. 9.0 ± 1.1 s, *p* < 0.05), shots per rally (11.1 ± 2.2 vs. 9.8 ± 1.1, *p* < 0.05), and rest time between games (145.2 ± 8.8 vs. 128.7 ± 5.9 s, *p* < 0.05)	N/A	N/A
Abian-Vicen et al. (2)	The smash (29.1 ± 8.4 vs. 21.6 ± 9.5%, *p* < 0.05) and drive (6.3 ± 3.9 vs. 2.0 ± 2.7%, *p* < 0.05) were used more frequently in men's singles than in the women's singles as the last shot of a rally; the differences in the frequency distributions of the clear, net, or lob shots between men's and women's singles were not significant	Men's singles had significantly higher values (*p* < 0.05) of the following variables than women's singles: match duration (2,378.0 ± 387.9 vs. 1,696.1 ± 170.4 s), real-time played (613.7 ± 80.1 vs. 493.6 ± 70.2 s), and total points played (68.0 ± 6.7 vs. 62.6 ± 4.9)	N/A	N/A
Abian-Vicen et al. (40)	N/A	N/A	N/A	The differences between the second round and quarterfinals in terms of sweat rate (1.04 ± 0.62 vs. 0.98 ± 0.43 L/h), rate of fluid intake (0.69 ± 0.26 vs. 0.91 ± 0.52 L/h), and dehydration levels (0.47 ± 1.03 vs. 0.23 ± 0.43%) were not significant; urinary protein concentration was significantly higher in the pre-game than in the post-game in both the second round (2.2 ± 7.5 vs. 34.6 ± 55.4) and quarterfinals (14.3 ± 21.3 vs. 51.9 ± 50.4)
Bisschoff et al. (45)	N/A	N/A	N/A	During the match, the average HR was 166.76 ± 13.84 bpm and maximum HR was 192.78 ± 11.31 bpm; in successful players, percentage HR variability of very low band peak frequencies relative power (*p* < 0.05), HR variability of the ratio of natural logarithmic transformation of low band peak frequencies and HR variability of high band peak frequencies relative power expressed as normalised units (*p* < 0.02) were significantly higher than those of less successful players; percentage HR variability of high band peak frequencies relative power (*p* < 0.01), HR variability of the natural logarithmic transformation of high band peak frequencies relative power expressed as normalised units (*p* < 0.02), and HR variability of low band peak frequencies in Hz (*p* < 0.02) were significantly lower for the successful player group than those for the less successful player group
Bisschoff et al. (44)	N/A	N/A	N/A	The average and maximum HR during the match were 167 ± 14 and 193 ± 11 bpm, respectively; urine shade scale results were moderate (2.76 ± 1.08) and muscle soreness tended to move to the higher side (1.74 ± 0.68); sleep quality was quite high (3.57 ± 1.03: 6.64 ± 1.38 h), and on average, players rated vigour as the most predominant mood state with a score of 11.08 ± 2.96; the correlation between recovery indicators and HR variability related variables was strong and significant (0.96, *p* = 0.014)
Chiminazzo et al. (49)	The serve (84.9 ± 16.9 vs. 74.0 ± 16.2, *p* < 0.02), net (361.2 ± 121.3 vs. 229.4 ± 79.4, *p* < 0.01), and smash shots (115.3 ± 31.8 vs. 96.2 ± 32.7, *p* < 0.03) were significantly more common in the playoffs than in the group stage	Match duration (3,464.0 ± 1,136.0 vs. 2,522.0 ± 721.0 s, *p* < 0.01), total rest time (2,557 ± 829.2 vs. 1,845.0 ± 560.4 s, *p* < 0.01), total points played (84.9 ± 16.9 vs. 74.1 ± 16.2, *p* < 0.02), shots per rally (11.5 ± 2.2 vs. 10.1 ± 1.7, *p* < 0.04), and total shots (1,001.0 ± 349.3 vs. 747.6 ± 205.1, *p* < 0.04) were significantly higher in the playoffs than in the group stage; rally time (9.3 ± 14.3 vs. 10.7 ± 8.7 s, *p* < 0.01, ES = 0.12) and rest time (25.6 ± 21.4 vs. 30.5 ± 23.2 s, *p* < 0.01, ES = 0.23) were significantly lower in the group stage than in the play-offs	N/A	N/A
Gawin et al. (43)	N/A	The total match duration was 0:49:54 ± 0:19:20 h, real-time played was 26.5 (21.4–31.7%), rally time was 9.3 ± 1.5 s, rest time was 23.1 ± 3.9 s, shots per rally were 4/2 (1–28), and shots per second was 0.56 ± 0.03 n/s; in both singles category (men's and women's), the rallies were shorter than those in the men's and mixed doubles (*p* < 0.001)	N/A	N/A
Gómez et al. (58)	N/A	The number of shots were higher during game 3rd than that during games 1st and 2nd (*p* = 0.001); rallies were longer during game 3rd than those during games 1st and 2nd (*p* = 0.001); the knockout stage was longer than the group stage (*p* = 0.006); and rest time in the knockout stage was longer than that during the group stage (*p* = 0.04); the frequency of shots was higher during the group stage than that during the knockout stage (*p* = 0.027); standard entropy increased as the second half (11 to 21 points) progressed in the first (group ACF: 0.67, knockout ACF: 0.54), second (knockout ACF: 0.58), and third (knockout ACF: 0.43) games	N/A	N/A
Phomsoupha et al. (50)	There were no significant differences in technical-tactical action variables in any session: clear (*p* = 0.857), drop (*p* = 0.794), smash (*p* = 0.654), and net shots (*p* = 0.728)	The average rally time, rest time between rallies, and effective playing time were 5.81 ± 0.32 s, 8.04 ± 0.35 s, and 41.54 ± 1.43%, respectively; there was a strong correlation between rally duration and recovery time (*r* = 0.742, *p* < 0.001) and a moderate correlation between shots frequency and rally duration (*r* = 0.504, *p* < 0.001)	N/A	The average HR was 168.3 ± 13.2 bpm (85% HR maximum); the correlation between HR and shots frequency was strong (*r* = 0.884, *p* < 0.001); blood lactate level increased from an initial value of 1.62 ± 0.43 to 6.87 ± 6.33 mmol/L after 10 min of play (*p* < 0.001)
Torres-Luque et al. (51)	N/A	The difference in match duration (*p* < 0.001) between the group stage (43:81 ± 12:10 min) and eliminatory phase (58:76 ± 18:75 min) was significant; the average longest rally duration in the group stage and eliminatory phase was 43:11 ± 18:04 s and 45:30 ± 10:24 s; rally times in the group stage and eliminatory phase were 9.53 ± 2:58 s and 10.23 ± 1:88 s, respectively; in Set 1st, values of the following variables were significantly higher in the eliminatory phase than in the group stage: duration of set (21:61 ± 5:37 vs. 18:13 ± 4:09, *p* < 0:001) and longest rally (42:30 ± 11:44 vs. 35:27 ± 13:02 s, *p* < 0:001)	N/A	N/A
Torres-Luque et al. (52)	N/A	In the Rio 2016 and London 2012 Olympics Games, the average match durations were 58.76 ± 18.75 vs. 55.87 ± 15.68 min, longest rally was 45.30 ± 10.24 vs. 42.68 ± 12.06 s, highest number of rally shots were 42.76 ± 9.04 vs. 42.00 ± 9.88, rally time was 10.23 ± 1.88 vs. 10.12 ± 2.29 s, and average number of rally shots were 8.92 ± 1.57 vs. 8.25 ± 1.70, respectively; in the Rio Olympics Games, the longest rally was in Sets 1st (42.30 ± 11.44 s) and 3rd (48.00 ± 7.74 s) (*p* < 0.01), and Set 3rd (*p* < 0.01) was longer in duration (29.40 ± 3.80 min) in Rio than in London Olympics Games (25.60 ± 2.63 min)	N/A	N/A
Abdullahi et al. (3)	With regard to shots-related variables, the drive (122.1 ± 27.4, 0.08 ± 0.02) and clear (118.0 ± 32.4, 0.08 ± 0.02) shots were the most common, followed by the serve (68.5 ± 12.8, 0.05 ± 0.01), smash (56.2 ± 23.1, 0.04 ± 0.01), and net shots (54.3 ± 19.7, 0.04 ± 0.01); with regard to foot movement-related variables, the chasse-step (174.6 ± 73.6, 0.12 ± 0.04) and shuffle foot (161.7 ± 66.1, 0.11 ± 0.04) movements were the most common; highly significant correlations were observed between chasse-step foot movements and smash shots (*r* = 0.71, *p* < 0.05) and between backward lunges and net shots (*r* = 0.71, *p* < 0.05)	The average match duration was 1,470.4 ± 341.9 s, real-time played was 432.9 ± 91.6 s, and the percentage of real-time played was 29.8 ± 4.5%; the average rally time was 5.6 ± 5.8 s, with an average of 6.5 ± 1.3 shots per rally during each match, and the average work density during matches was 0.4 ± 0.1 work/rest; players rested for 17.3 ± 4.6 s in-between rallies during each match	N/A	N/A
Abdullahi et al. (5)	N/A	N/A	The average distance cover was 1,763 ± 751.4 m; the furthest distance covered was the low-intensity distance (978.09 ± 331 m), followed by the medium-intensity (616 ± 387 m) and high-intensity (170.07 ± 134.72 m) distances; moderately significant correlations were observed between absolute distance (*r* = 0.42, *p* < 0.05) covered and time spent in the high-intensity zone (*r* = 0.44, *p* < 0.05)	During match-play, the minimum HR was 91.2 ± 17.4 bpm, average HR was 157.1 ± 13.9 bpm, and maximum HR was 188.73 ± 11.7 bpm; the average PL was 187 ± 79.6 (5.3 ± 1.1/s, 5.3 ± 1.1/min) and average peak PL was 10.8 ± 13.9 (0.01 ± 0.01/s); the correlation between PL and HR in the high-intensity zone was moderately significant (*r* = 0.44, *p* < 0.05)
Abián-Vicén et al. (38)	N/A	N/A	N/A	The sweat rate during the badminton match was 1.14 ± 0.46 L/h and rate of fluid intake was 1.10 ± 0.55 L/h; there was a significant loss of body mass during the match (pre-match: 74.4 ± 7.2 kg, post-match: 74.1 ± 7.2, *p* < 0.05)—with dehydration 0.32 ± 0.83% in the former and 0.37 ± 0.50% in the latter—and a significant decrease in the urinary pH after the match (pre: 7.20 ± 1.08, post: 6.28 ± 1.05, *p* < 0.05); the post-match nitrite (pre: 0.4%, post: 52.2%) and protein concentrations (pre: 8.6%, post: 60.9%) were significantly higher (*p* < 0.05) than those were pre-match
Faude et al. (37)	N/A	N/A	N/A	The average VO_2_ was 46.0 ± 4.5 ml/kg/min, HR was 166 ± 6 bpm, blood lactate level was 1.9 ± 0.1 mmol/L, RER was 0.99 ± 0.06, V_E_ was 94.3 ± 6.4 L/min^−1^, b_f_ was 47.4 ± 5.9 min^−1^, and EE was 68.0 ± 7.5 kJ/min; men's singles had higher values (*p* < 0.05) of VO_2_ (46.0 ± 4.5 vs. 36.4 ± 2.8), V_E_ (94.3 ± 6.4 vs. 61.1 ± 8.7), and EE (68.0 ± 7.5 vs. 45.9 ± 6.7) than women's singles; players who won and those who lost matches did not differ significantly (*p* > 0.41) in terms of VO_2_ and HR
Hoffmann et al. (65)	N/A	The year affected all variables from 2006 to 2017, except for the total points played. Concretely, game duration, rally time, rest time, rest time at point 11 and rest time between games increased by 54.0% (*p* = 0.002), 62.2% (*p* = 0.000), 49.3% (*p* = 0.000), 44% (*p* < 0.001) and 74.9% (*p* = 0.000), respectively	N/A	N/A
Jiménez et al. (39)	N/A	N/A	N/A	Regarding sex influenced testosterone levels (*p* = 0.0001), men's singles had higher testosterone levels than women's singles before the competition (*p* = 0.007); in men's singles, testosterone level rose in winners (*p* < 0.0001 and *p* = 0.019) and dropped in losers (*p* < 0.0001 and *p* = 0.016); after the competition, cortisol levels were higher in losers (*p* < 0.0001 in men's singles); however, there was no variation in winners (*p* > 0.9 in men's singles)
Le Mansec et al. (62)	N/A	There was no significant main effect among five playing categories on the duration of the match (average: 42.0 ± 11.6 min); for rally duration, men's singles were longer (*p* < 0.001) than men's doubles and mixed doubles; for effective playing time, men's singles were greater (*p* < 0.001) than men's doubles, women's doubles, and mixed doubles; for shots per second, men's singles were greater (*p* < 0.001) than women's singles, men's doubles, and mixed doubles	N/A	N/A
Leong et al. (46)	N/A	The differences between professional and junior players in terms of mean match duration (1,449.2 ± 434.6 vs. 1,066.3 ± 152.0 s, *p* < 0.001), number of shots per rally (12.3 ± 8.6 vs. 8.2 ± 5.9, *p* < 0.001), real-time played (419.9 ± 101.9 vs. 306.7 ± 62.72 s, *p* < 0.001), and rally duration (11.9 ± 8.04 vs. 8.1 ± 5.3 s, *p* < 0.001) were significant	N/A	N/A
Lin et al. (61)	N/A	N/A	The total number of lunges per player in a match was 160–240 (average, 194 ± 18); the knee extension MVC torque decreased significantly (by 12.7 ± 2.9% from 278.4 ± 50.8 Nm before the match, *p* < 0.05); knee extensor voluntary activation in the dominant leg decreased significantly (*p* < 0.05) from before (90.4 ± 1.9%) to after (80.0 ± 2.2%) matches	Average VO_2_, HR, RPE, and pre-match and post-match blood lactate level were 44.3 ± 8.6 ml/kg/min (80% of VO_2max_), 162.0 ± 10.6 bpm, 84% of maximum HR, 7.0 ± 2.0, and 1.8 ± 0.3 mmol/L, 7.2 ± 1.3 mmol/L, respectively
Valldecabres et al. (48)	The most common shots were net shots (36.09%), and men's singles showed a higher use of smashes (11.46%) and lobs (22.08%) shots; in men's or women's singles, the smash was the most successful shot, and net shots were least successful	The values of total real-time played (880.473 vs. 772.564 s), average rally time (12.061 vs. 10.033 s), shots per rally (6.452 vs. 5.403), average rest time (45.550 vs. 36.591 s), and total shots (471 vs. 416) were all higher for men's singles than those for women's singles	N/A	N/A
Fernandez-Fernandez et al. (41)	N/A	The average match duration was 1,411 ± 422 s and average effective playing time was 36.6 ± 4.3%; men's singles had higher rally duration (6.8 ± 4.8 vs. 5.7 ± 3.1 s, *p* < 0.05, ES = 0.81), rest time between rallies (10.5 ± 8.8 vs. 8.8 ± 7.2 s, *p* < 0.05, ES = 0.81), and shots per rally (6.4 ± 4.8 vs. 4.7 ± 2.8, *p* < 0.001, ES = 1.56) values than women's singles	N/A	Internal load variable: HR: 170 ± 9 bpm, blood lactate levels: 3.2 ± 1.8 mmol/L, RPE: 14.6 ± 1.8; the differences between men's and women's singles in terms of any internal load variables were not significant (all *p* values >0.05, ES: 20.33–0.08)
Fu et al. (57)	N/A	N/A	There were no significant differences between men's and women's singles in terms of decelerations (46.38 ± 29.91 vs. 68.17 ± 29.86), change of direction, left (144.13 ± 40.76 vs. 165.67 ± 75.82), changes of direction, right (82.25 ± 35.25 vs. 70.00 ± 11.54), or jumps (27.63 ± 16.17 vs. 31.50 ± 19.58) or between the victorious and defeated players in terms of accelerations, decelerations, changes of direction (left or right), or jumps	The average glycolytic system contribution was 13.62 ± 11.04 kJ, aerobic energy contribution was 832.07 ± 175.63 kJ, total energy contribution was 920.82 ± 164.26 kJ, and average rate of lipid oxidation was 0.67 ± 0.16 g/min; the average HR was 162.38 ± 18.35 bpm, maximum HR was 194.50 ± 15.00 bpm, absolute PL was 111.85 ± 19.77 AU, and relative PL was 5.02 ± 0.26 AU; men's singles had higher anaerobic lactic capacity (45.04 ± 10.04 vs. 28.55 ± 5.14 kJ, *p* = 0.008) and average rate of carbohydrate oxidation (1.56 ± 0.69 vs. 0.96 ± 0.12 g/min, *p* = 0.044) than women's singles
Green et al. (60)	N/A	Rally duration (6.1 ± 3.9 vs. 5.3 ± 3.5 s, *p* = 0.005), shots per rally (5.9 ± 3.5 vs. 5.3 ± 3.7, *p* = 0.012), work density (0.53 ± 0.17 vs. 0.48 ± 0.23 work/rest, *p* < 0.001), and effective playing time (33.8 ± 7.4 vs. 30.9 ± 9.0%, *p* < 0.001) were higher in the second game than in the first game	N/A	During match-play, the average HR was 151 ± 12 bpm, blood lactate levels during breaks was 2.42 ± 0.44 mmol/L, post-match blood lactate level was 3.33 ± 0.83 mmol/L, RER was 0.84 ± 0.07, EE was 46.7 ± 4.4 kJ/min, and VO_2_ was 39.2 ± 3.9 ml/kg/min (62% of VO_2max_ during the 20-m shuttle run test)
Kui et al. (Wei Sheng (63)	The shots with the highest win rates were the smash (men's singles number 1: 42.50%, number 2: 28.57%) and net shots (men's singles number 1: 17.5%, number 2: 22.86%)	Average activity profile data: average shot frequency: 0.99 ± 0.05 s, average shots per rally: 7.28 ± 1.14, total match duration: 48.15 min, average rally duration: 6.98 ± 1.09 s, and average time of rest: 20.92 ± 3.77 s	N/A	Average HR: men's singles number 1 (Set 1st: 114, Set 2nd: 153, Set 3rd:152 bpm), number 2: (Set 1st: 112, Set 2nd: 150, Set 3rd: 149 bpm)
Rojas-Valverde et al. (56)	N/A	N/A	Average relative distance: 46.23 ± 3.73 m/min (1st game), 45.36 ± 2.90 m/min (2nd game), 42.55 ± 3.82 m/min (3rd game), and 44.13 ± 3.99 m/min (4th game); average relative acceleration: 26.33 ± 2.01 N/min (1st game), 26.38 ± 1.75 N/min (2nd game), 25.53 ± 1.93 N/min (3rd game), and 25.26 ± 1.98 N/min (4th game, average maximum acceleration: 3.84 ± 0.36 (1st game), 3.88 ± 0.32 (2nd game), 3.89 ± 0.33 (3rd game), and 3.85 ± 0.32 (4th game); and average maximum speed: 10.84 ± 1.46 km/h (1st game), 10.81 ± 1.44 km/h (2nd game), 10.74 ± 0.99 km/h (3rd game), and 11.03 ± 1.74 km/h (4th game); relative and maximum accelerations were significantly higher (*p* = 0.01) for men than women players	Average HR: 1st game, 172 ± 8.92 bpm; 2nd game, 177.92 ± 10.09 bpm; 3rd game, 174.46 ± 9.77 bpm; and 4th game, 178.55 ± 8 bpm. There was no significant interaction (sex vs. games) for the average HR as an internal load variable
Sales et al. (59)	N/A	N/A	N/A	During official matches, the men's singles remained in zones 4 (at 80%–90% maximum HR) and 5 (at 90%–100% maximum HR) longer than in zone 1 (at <60% maximum HR) (*p* < 0.05, *η*^2^ = 0.52); men's and women's singles did not differ in terms of zone HR during official (*p* = 0.4, *η*^2^ = 0.22), and simulated matches (*p* < 0.05, *η*^2^ = 0.22)
Deka et al. (47)	N/A	N/A	The total step count was 2,404 ± 360; the mean numbers of steps during the first and second 15 min of match-play did not differ significantly, with 1,264 ± 176 and 1,140.7 ± 206, respectively	The mean VO_2_ was 34.4 ± 5.8 ml/kg/min, metabolic equivalent was vigorous intensity (9.8 METS), and average HR during match-play was 166.2 ± 9.23 bpm; the HR in the last 10 min was significantly higher than that in the first 10 min (*p* < 0.001); blood lactate levels and RPE were significantly higher at 15 and 30 min (*p* < 0.001)
Women's singles category				
Abian-Vicen et al. (2)	Drop shots (3.8 ± 3.5 vs. 9.0 ± 6.0%, *p* < 0.05) were used more frequently by the women's singles; unforced errors were more frequent in women's singles than in men's singles (48.6 ± 9.0% vs. 41.0 ± 9.4%, *p* < 0.05)	The percentage of time played was higher for women's singles (*p* < 0.05) than that for men's singles; rallies were significantly more frequent (*p* < 0.05) between 3 and 6 s; comparing the course of each game, women's singles had higher values for work density (game 1st: 0.45 ± 0.05, game 2nd: 0.44 ± 0.04 work/rest vs. game 1st: 0.38 ± 0.06, game 2nd: 0.36 ± 0.04 work/rest) than men's singles (*p* < 0.05)	N/A	N/A
Abian-Vicen et al. (40)	N/A	N/A	N/A	There were no significant differences between the second round and quarterfinal matches in terms of sweat rate (1.04 ± 0.62 vs. 0.98 ± 0.43 L/h), rate of fluid intake (0.69 ± 0.26 vs. 0.91 ± 0.52 L/h), and dehydration level (0.47% ± 1.03 vs. 0.23 ± 0.43%), respectively; pre-game urinary protein concentration was significantly higher than post-game urinary protein concentration in both the second round (2.2 ± 7.5 vs. 34.6 ± 55.4) and quarterfinals (14.3 ± 21.3 vs. 51.9 ± 50.4)
Gawin et al. (43)	N/A	Total match duration: 0:47:28 ± 0:16:35 h, performance time: 29.2 (24.1–32.9%), rally time: 9.2 ± 1.4 s, rest time: 19.4 ± 3.4 s, and shots per rally: 4/2 (1–21); the lowest value of shots per second was observed in the women's singles (0.49 ± 0.02); in both singles category (men's and women's), the rallies were shorter than those in the men's doubles and mixed doubles (*p* < 0.001, *r* = 0.81)	N/A	N/A
Gómez-Ruano et al. (54)	Approximately 49.6% of the points were won by the serving player; the type of serve was not significantly (*p* > 0.05) associated with winning the point when serving; opponents showed significant relationships (*p* < 0.001) between type of serve and winning the point serving when using the forehand flick and forehand short serve	Match duration was 41.8 min, set 1 duration was 22.1 min, set 2 duration was 20.7 min, set 3 duration was 41.8 min, rally time was 7.87 s, rest time was 22.1 s, shots per rally were 8.0, and frequency was 1.01 s; in 27.3% of cases, medallists played the rally, which lasted 12.3 s and had a shots frequency of 0.98	N/A	N/A
Torres-Luque et al. (51)	N/A	The differences (*p* < 0.001) between the group stage and eliminatory phase in terms of match duration (40:11 ± 11:88 vs. 50:66 ± 13:75 min), average rally shots (6.64 ± 1.40 vs. 7.58 ± 1.28), and shuttles used (9:59 ± 3:16 vs. 14:83 ± 6:22) were significant; in Set 1st, the eliminatory phase had higher values than the group stage for the following variables: duration of the set (21:58 ± 4:32 vs. 17:76 ± 3:92 min, *p* < 0.001), longest rally shots (31:00 ± 9:99 vs. 25:52 ± 7:26, *p* < 0,02), and average rally shots (7:83 ± 1:43 vs. 6:97 ± 1:51, *p* < 0.001)	N/A	N/A
Torres-Luque et al. (52)	N/A	In the Rio 2016 and London 2012 Olympics Games, the average match duration was 50.66 ± 13.75 vs. 48.92 ± 14.62 min, longest rally was 38.50 ± 7.37 vs. 31.71 ± 13.15 s, number of shots in the longest rally was 34.16 ± 9.22 vs. 32.07 ± 7.26, and the average number of shots in a rally was 7.58 ± 1.28 vs. 7.07 ± 1.30; the length of rallies in a match was longer (*p* < 0.05) in Rio (10.50 ± 1.74 s) than in London (8.71 ± 2.94 s); the Rio Olympic Games had a longer duration of all sets (*p* < 0.05) and a higher number of shots per rally than London Olympic Games (*p* < 0.05)	N/A	N/A
Xiang-Qian Xu et al. (64)	N/A	The average results of the women's singles players (game duration, longest rally, average rally and total points played) particularly in the elimination phase, are higher (*p <* 0.020) than the average results of the group stage	N/A	N/A
Abián-Vicén et al. (38)	N/A	N/A	N/A	Sweat rate during a badminton match was 1.02 ± 0.61 L/h; the reduction in body mass between the pre-match and post-match conditions was significant (pre: 60.7 ± 4.1, post: 60.5 ± 4.1 kg, *p* < 0.05), with dehydration of 0.32 ± 0.83% in the former and 0.37 ± 0.50% in the latter; compared with before the match, after the match urinary pH values were significantly reduced (pre: 7.2061.21, post: 6.2560.87, *p* = 0.059); contrarily, nitrite (pre: 0.0%, post: 58.3%) and protein concentrations (pre: 10.0%, post: 66.7%) increased significantly (*p* < 0.05)
Faude et al. (37)	N/A	N/A	N/A	The average VO_2_ was 36.4 ± 2.8 ml/kg/min, HR was 170 ± 10 bpm, blood lactate level was 1.9 ± 0.9 mmol/L, RER was 0.99 ± 0.08, V_E_ was 61.1 ± 8.7 L/min^−1^, b_f_ was 44.9 ± 5.9 min^−1^, and EE was 45.9 ± 6.7 kJ/min; women's singles had lower (*p* < 0.05) values of VO_2_ (36.4 ± 2.8 vs. 46.0 ± 4.5), V_E_ (61.1 ± 8.7 vs. 94.3 ± 6.4), and EE (45.9 ± 6.7 vs. 68.0 ± 7.5) than men's singles; players who won vs. those who lost matches did not differ HR
Jiménez et al. (39)	N/A	N/A	N/A	In women’ singles, regarding sex influenced testosterone levels (*p* = 0.0001), testosterone levels rose in winners (*p* = 0.019) and dropped in losers (*p* = 0.016); after the competition, cortisol levels rose in losers (*p* = 0.005), whereas there was no variation in winners (*p* > 0.6)
Le Mansec et al. (62)	N/A	There was no significant main effect among five playing categories on the duration of the match (average: 42.0 ± 11.6 min); for effective playing time, women's singles were greater (*p* < 0.001) than men's doubles, women's doubles, and mixed doubles; for shots per second, women's singles were greater (*p* < 0.001) than men's doubles, women's doubles, and mixed doubles	N/A	N/A
Valldecabres et al. (48)	The most commonly used shots were net shots (28.13%); the use of the drive (8.89%) and drop shots (12.02%) were more common among women's singles; in both women's and men's singles category, the smash was the most successful shot, and net shots were the least successful	Work density (0.274 vs. 0.265 work/rest), shot frequency (0.538 vs. 0.535), and rest time between games 1–2 (163.320 vs. 152.424 s) were all greater in the women's singles than in men's singles category	N/A	N/A
Zhang et al. (66)	Overhead shots of two right-handers’ games were significantly higher than those of opposite handedness's games with a small effect size (*p* < 0.05, ES = 0.492)	N/A	N/A	N/A
Fernandez-Fernandez et al. (41)	N/A	Average total match time was 1,026 ± 108 s, effective playing time was 39.2 ± 3.5%, rally duration was 5.7 ± 3.1 s, rest time between rallies was 8.8 ± 7.2 s, and shots per rally were 4.7 ± 2.8	N/A	Average internal load variable values: HR: 174 ± 7 bpm, blood lactate level: 2.5 ± 1.3 mmol/L, and RPE: 14.2 ± 1.9; the differences between men's and women's singles in any internal load variables were not significant (all *p* values > 0.05, ES: 20.33–0.08)
Fu et al. (57)	N/A	N/A	Compared to men's singles, women's singles showed greater acceleration (72.50 ± 18.63 vs. 41.75 ± 14.92 N, *p* = 0.005); there were no significant differences between men's and women's singles in terms of decelerations (46.38 ± 29.91 vs. 68.17 ± 29.86 N), changes of direction, left (144.13 ± 40.76 vs. 165.67 ± 75.82 N), changes of direction, right (82.25 ± 35.25 vs. 70.00 ± 11.54 N), and jumps (27.63 ± 16.17 vs. 31.50 ± 19.58 N) or between victorious and defeated players in terms of accelerations, decelerations, changes of direction (left or right), and jumps	The average ATP-PCr system contribution was 28.55 ± 5.14 kJ, glycolytic system contribution was 7.30 ± 3.19 kJ, aerobic energy contribution was 826.76 ± 226.96 kJ, total energy contribution was 862.62 ± 224.83 kJ, average rate of carbohydrate oxidation was 0.96 ± 0.12 g/min, and average rate of lipid oxidation was 0.52 ± 0.08 g/min; the mean HR was 171.17 ± 8.93 bpm, maximum HR was 198.50 ± 4.76 bpm, and relative PL was 4.92 ± 0.86 AU; women's singles showed greater workloads in terms of absolute PL than men's singles (147.82 ± 31.24 vs. 111.85 ± 19.77, *p* = 0.029)
Kui et al. (63)	The clear (40.54%) and drop (38.5%) shots were the most common	Average activity profiles: average shot frequency: 0.82 ± 0.06 s, average shot per rally: 6.72 ± 1.41, total match duration: 50.86 min, average rally time: 8.13 ± 1.15 s, and average duration of rest: 14.30 ± 3.61 s	N/A	Average HR in women's singles number 1: Set 1st: 147, Set 2nd: 179, and Set 3rd: 180 bpm and in women's singles number 2: Set 1st: 130, Set 2nd: 136, Set 3rd: 157 bpm
Nagano et al. (55)	N/A	N/A	The top five movements were lunging with the dominant hand-side leg during an underhand stroke, landing the non-dominant hand-side leg after an overhand stroke, landing on the dominant hand-side leg after an overhand stroke, cutting from a split step using the non-dominant hand-side leg, and cutting from a split step using the dominant hand-side leg; overhand stroke landings on the dominant leg resulted in greater acceleration than other movements (*p* < 0.001); lunging with the dominant hand-side leg during an underhand stroke involved lesser vertical acceleration than the other movements (*p* < 0.001); and lunging with the dominant hand-side leg during an underhand stroke involved greater anteroposterior acceleration than landing on the dominant and non-dominant hand-side legs after an overhand stroke and cutting from a split step using the non-dominant hand-side leg (*p* < 0.001)	N/A
Rojas-Valverde et al. (56)	N/A	N/A	The average relative distances were 44.54 ± 4.46 m/min (1st game), 44.01 ± 5.69 m/min (2nd game), 42.75 ± 5.31 m/min (3rd game), and 42.85 ± 5.39 m/min (4th game); average relative accelerations were 24.77 ± 2.36 n/min (1st game), 24.41 ± 1.76 n/min (2nd game), 24.57 ± 1.81 n/min (3rd game), and 24.96 ± 1.86 n/min (4th game); average maximum accelerations were 3.41 ± 0.37 (1st game), 3.48 ± 0.42 (2nd game), 3.43 ± 0.38 (3rd game), and 3.54 ± 0.39 (4th game); and average maximum speeds were 10.60 ± 1.65 km/h (1st game), 10.36 ± 1.37 km/h (2nd game), 10.68 ± 0.97 km/h (3rd game), and 11 ± 2.15 km/h (4th game)	The average HR of women's singles during the 1st, 2nd, 3rd, and 4th games was 172.68 ± 11.86, 175 ± 11.59, 172.85 ± 15.71, and 172.15 ± 22 bpm, respectively; there was no significant interaction (sex vs. games) in average HR as an internal loads’ variable
Sales et al. (59)	N/A	N/A	N/A	Women's singles spent more time in zones 3 (at 70%–80% of the maximum HR) and 4 (at 80%–90% of the maximum HR) than in zones 1 (at <60% HR maximum), 2 (at 60%–70% the maximum HR), and 5 (at 90%–100% the maximum HR) during the simulated match (*p* = 0.006); women's singles spent more time in zone 5 than that in zones 1, 2, and 3 during official matches (*p* < 0.02); EE was 10.74 ± 0.53 kcal min^−1^; no difference was observed between women's and men's singles in terms of zone HR during official (*p* = 0.4, *η*^2^: 0.22) and simulated matches (*p* < 0.05, *η*^2^: 0.22)

ACF, autocorrelation function; ATP-PCr, adenosine triphosphate phosphocreatine; AU, arbitrary units; b_f_, breathing frequency; BPM, beats per minutes; EE, energy expenditures; ES, effect sizes; g/min, gram per minute; HR, heart rate; Hz, hertz; h, hours; kg, kilogram; kJ/min, kilojoule per minute; kJ, kilojoule; km/h, kilometre per hour; L/h, liters per hour; MVC, maximal voluntary isometric contraction; METS, metabolic equivalents; m, meter; min, minutes; m/min, meter per minute; mmol/L, millimole per liter; ml/kg/min, millilitres per minute per kilogram; N, newton; N/A, not available; Nm, newton per meter; N/min, newton per minute; n/s, number per seconds; *η*^2^, partial eta squared; pH, potential of hydrogen; PL, player loads; r, correlation volume; RER, respiratory exchange ratio; RPE, rate of perceived exertion; s, seconds; V_E_, minute ventilation; VO_2_, oxygen uptake; VO_2max_, oxygen uptake maximum.

**Table 5:** Study characteristics of the 34 included studies concerning the match-play outcome measures of the doubles category in both sexes and mixed.

Authors	Outcome (technical-tactical actions)	Outcome (activity profiles)	Outcome (external loads)	Outcome (internal loads)
Men's doubles category				
Abián-Vicén et al. (4)	N/A	Average match duration was Beijing: 2,657.0 ± 755.7, London: 2,903.8 ± 859.7, Rio: 3,900.4 ± 899.2 s; average real-time played was Beijing: 478.3 ± 153.8, London: 569.9 ± 135.2, Rio: 616.3 ± 146.9 s; average rest time between games was Beijing: 135.4 ± 8.3, London: 132.9 ± 8.8, Rio: 147.4 ± 17.6 s, and average work density was Beijing: 0.25 ± 0.06, London: 0.28 ± 0.06, Rio: 0.21 ± 0.03 work/rest; the number of shots-per rally was higher in London than in Beijing (*p* = 0.039, ES = 1.6) higher values were recorded in the shortest intervals (0–3 and 3–6 s)	N/A	N/A
Gawin et al. (43)	N/A	The total match duration was 0:45:55 ± 0:16:27 h, performance time was 20.4 (17.2–24.5%), rally time was 6.7 ± 1.5 s, rest time was 23.3 ± 3.7 s, shots per rally was 4/2 (1–34), and shots per second was 0.76 ± 0.03 n/s; no statistically significant differences was found in average resting times among the playing categories (*p* = 0.10); the difference in shots per second between men's doubles and mixed doubles was not significant (0.76 ± 0.03 vs. 0.72 ± 0.03, respectively)	N/A	N/A
Torres-Luque et al. (51)	N/A	All match-related variable values were higher in the eliminatory phase than in the group phase (*p* < 0.05); the average match duration in the group stage and eliminatory phase was 48:68 ± 17:87 and 68:94 ± 11:76 min, average of the longest rally in the group stage and eliminatory phase was 42:30 ± 19:96 and 33:94 ± 10:20 s, and the average rally in the group stage and eliminatory phase was 6:70 ± 2:16 and 7:23 ± 1:67 s; men's doubles had longer matches as well as sets (set 1st: 23:00 ± 5:63 and set 2nd: 29:94 ± 11:36), and average rallies (set 1st: 7:70 ± 1:31 and set 3rd: 6:66 ± 0:98)	N/A	N/A
Apriantono et al. (53)	A total of 350 rallies in three matches; the drive shot was most common (466 shots), followed by the drop (337 shots) and lob shots (298 shots)	N/A	N/A	Blood lactate: pre-match (after warming-up): 3.05 ± 1.13 mmol/L, post-match: 4.6 ± 1.11 mmol/L
Le Mansec et al. (62)	For all types of doubles, the player who played the most shuttlecocks in the rear part of the court performed more smash shots than his/her partner (*p* < 0.05); in men's doubles, player who played the most shuttlecocks in the rear part of the court performed a lesser proportion of nets than his partner (*p* < 0.01)	There was no significant main effect among five playing categories on the duration of the match (average: 42.0 ± 11.6 min); for effective playing time, men's doubles were greater (*p* < 0.001) than women's doubles	N/A	N/A
Women's doubles category				
Abián-Vicén et al. (4)	N/A	The average match durations was Beijing: 2,840.6 ± 652.7, London: 559.5 ± 884.3, Rio: 4,037.4 ± 1,053.9 s; average of the real time played was Beijing: 767.7 ± 242.2, London: 608.5 ± 213.2, Rio: 913.5 ± 240.5 s; average of rest time between games was Beijing: 148.3 ± 19.2, London: 135.1 ± 10.5, Rio: 147.6 ± 10.7 s; and average work density was Beijing: 0.41 ± 0.07, London: 0.36 ± 0.06, Rio: 0.34 ± 0.07 work/rest; the percentage of time played was higher in the women's doubles than that in the men's doubles in the three Olympics Games (Beijing: 95% CI: 5.5–11.8%, *p* < 0.001, ES = 2.6, London: 95% CI: 0.7%–7.0%, *p* = 0.016, ES = 1.4, Rio: 95% CI: 4.0–10.2%, *p* < 0.001, ES = 2.4). in all the Olympics Games analysed, work density was higher (*p* < 0.001) and shot frequency was lower (*p* < 0.001) in women's doubles	N/A	N/A
Gawin et al. (43)	N/A	The total match duration was 0:40:04 ± 0:10:40 h, performance time was 30.1 (23.4–37.2%), rally time was 10.1 ± 3.2 s, rest time was 20.0 ± 5.3 s, shots per rally were 4/2 (1–41), and shots per second were 0.62 ± 0.05, there were no statistically significant differences in average resting time among all playing categories (*p* = 0.10); shots per rally in women's doubles were higher than those in the men's doubles (*p* = 0.01), women's singles (*p* < 0.01), and mixed doubles (*p* < 0.01)	N/A	N/A
Torres-Luque et al. (51)	N/A	All match-related variable values were significantly higher in the eliminatory phase than in the group phase (*p* < 0.05); the average match duration in the group phase and eliminatory phase was 47:75 ± 13:00 and 68:62 ± 17:13 min; the average of the longest rally in the group phase and eliminatory phase was 54:33 ± 18:58 and 51:87 ± 13:77 s, and the average rally in the group phase and eliminatory phase was 10:33 ± 2:23 and 10:37 ± 1:99 s, respectively	N/A	N/A
Le Mansec et al. (62)	For all types of doubles, the player who played the most shuttlecocks in the rear part of the court performed more smash shots than his/her partner (*p* < 0.05); there was no difference between the player who played the most shuttlecocks in the rear part of the court and her partner of proportional net shots in women's doubles	There was no significant main effect among five playing categories on the duration of the match (average: 42.0 ± 11.6 min); for rally duration, women's doubles were greater (*p* < 0.001) than men's doubles and mixed doubles; for rest time, women's doubles were greater (*p* < 0.001) than mixed doubles; for effective playing time, women's doubles were greater (*p* < 0.001) than men's doubles and mixed doubles; for shots per second, women's doubles were greater (*p* < 0.001) than men's doubles and mixed doubles	N/A	N/A
Mixed doubles category				
Gawin et al. (43)	N/A	The total match duration was 0:40:33 ± 0:09:14 h, performance time was 19.4 (17.1–21.5%), rally time was 5.6 ± 0.5 s, rest time was 20.6 ± 3.2 s, shots per rally were 3/2 (1–23), and shots per second were 0.72 ± 0.03; the average resting time was not statistically significant in any playing category (*p* = 0.10); the difference in identical shots per second between mixed doubles and men's doubles was not significant (0.72 ± 0.03 vs. 0.76 ± 0.03 n/s)	N/A	N/A
Torres-Luque et al. (51)	N/A	All match-related variable values were significantly higher in the eliminatory phase than in the group phase (*p* < 0.05); the average match duration in the group stage and eliminatory phase were 47:45 ± 16:36 and 44:25 ± 6:19 min, the average of the longest rally in the group stage and eliminatory phase were 32:66 ± 10:97 and 37:00 ± 6:96 s, and average rally in the group stage and eliminatory phase were 7:58 ± 1:79 and 7:87 ± 1:20 s, respectively	N/A	N/A
Le Mansec et al. (62)	For all types of doubles, the player who played the most shuttlecocks in the rear part of the court performed more smash shots than his/her partner (*p* < 0.05); in mixed doubles, player who played the most shuttlecocks in the rear part of the court performed a lesser proportion of nets than his/her partner (*p* < 0.01)	There was no significant main effect among five playing categories on the duration of the match (average: 42.0 ± 11.6 min); for effective playing time, mixed doubles were greater (*p* < 0.001) than women's doubles; for shots per second, mixed doubles were greater (*p* < 0.001) than men's doubles	N/A	N/A

ES, effect sizes; h, hours; N/A, not available; min, minutes; mmol/L, millimole per liter; n/s, number per seconds; s, seconds; 95% CI, 95% confidence interval.

In terms of playing context, official matches were most commonly investigated in 24 studies (2–5, 38–40, 42–46, 48, 49, 51, 52, 54, 56–58, 62, 64–66), followed by simulated matches in nine studies (37, 41, 47, 50, 53, 55, 60, 61, 63). One study (59) analysed official and simulated matches. Regarding playing categories, men's singles were most often investigated in 28 studies (2, 3, 5, 37–52, 56–63, 65), followed by women's singles in 19 studies (2, 37–41, 43, 48, 51, 52, 54–57, 59, 62–64, 66). Fewer studies have been conducted in the doubles categories: men's doubles in five studies (4, 43, 51, 53, 62), women's doubles in four studies (4, 43, 51, 62), and mixed doubles in three studies (43, 51, 62). Concerning outcome measures, most studies focused on activity profiles with eight studies (4, 43, 46, 51, 52, 58, 64, 65), followed by the internal loads with seven studies (37–40, 44, 45, 59). Seven studies (2, 3, 42, 48, 49, 54, 62) combined technical-tactical actions and activity profiles, five studies (5, 47, 56, 57, 61) combined internal and external loads, two studies (41, 60) combined activity profiles and internal loads, two studies (50, 63) combined technical-tactical actions, activity profiles, and internal loads, one study (53) combined technical-tactical actions and internal loads, one study (66) focused on technical-tactical actions only, and one study (55) focused on external loads only.

### 3.4 Differences in match-play outcome measures

Table 6 summarises the descriptive differences in match-play outcome measures. The outcomes are further specified concerning sexes and playing categories.

**Table 6:** Descriptive overview of the match-play outcome measures according to the five playing categories in badminton (means and standard deviations).

Variables	Categories				
Technical-tactical actions	Men's singles	Women's singles	Men's doubles	Women's doubles	Mixed doubles
Net shots (%)	24.5 ± 7.5 (12.2–36.1)	23.3 ± 6.8 (18.5–28.1)	N/A	N/A	N/A
Drop shots (%)	12.7 ± 5.2 (3.8–16.9)	17.7 ± 13.9 (9.0–38.5)	N/A	N/A	N/A
Drive shots (%)	9.9 ± 11.9 (1.4–27.5)	4.6 ± 3.7 (2.0–8.9)	N/A	N/A	N/A
Clear shots (%)	16.4 ± 5.3 (7.3–26.6)	22.6 ± 12.3 (13.7–40.5)	N/A	N/A	N/A
Smash shots (%)	20.3 ± 9.4 (11.5–42.5)	14.6 ± 6.3 (9.4–21.6)	N/A	N/A	N/A
Activity profiles	Men's singles	Women's singles	Men's doubles	Women's doubles	Mixed doubles
Match duration (s)	2,364.2 ± 1,020.9 (1,066.3–4,047.0)	2,618.2 ± 716.6 (1,026.0–3,680.0)	3,106.2 ± 665.0 (2,405.0–4,174.0)	3,040.1 ± 743.0 (2,402.0–4,142.0)	2,653.0 ± 176.5 (2,433.0–2,865.0)
Rally time (s)	8.8 ± 2.0 (5.3–12.1)	9.3 ± 2.0 (5.7–13.9)	6.5 ± 0.7 (5.3–7.3)	9.7 ± 0.9 (7.7–10.4)	6.8 ± 1.1 (5.6–7.9)
Real-time played (s)	512.9 ± 208.9 (306.7–880.5)	555.4 ± 193.8 (400.0–772.6)	553.9 ± 57.4 (478.3–616.3)	752.7 ± 126.3 (608.5–913.5)	N/A
Effective playing time (%)	34.1 ± 5.3 (27.8–41.5)	29.0 ± 6.6 (20.9–39.2)	19.2 ± 2.3 (15.8–21.6)	27.2 ± 5.0 (22.9–35.1)	N/A
Longest rally (n)	41.7 ± 1.7 (39.2–42.8)	32.2 ± 4.1 (28.6–37.6)	42.9 ± 0.6 (42.4–43.3)	57.4 ± 1.4 (56.4–58.4)	39.0 ± 3.1 (36.8–41.3)
Shots per rally (n)	8.9 ± 2.1 (5.3–12.3)	6.9 ± 1.1 (4.7–8.6)	9.5 ± 1.3 (8.2–10.7)	11.8 ± 1.7 (9.8–12.9)	N/A
Shots per second (n/s)	1.0 ± 0.2 (0.6–1.1)	0.8 ± 0.2 (0.5–1.0)	1.5 ± 0.0 (1.4–1.5)	1.3 ± 0.1 (1.1–1.3)	N/A
Total points played (points)	77.0 ± 7.5 (68.0–84.9)	42.3 ± 20.6 (31.2–79.0)	88.1 ± 7.9 (79.4–98.5)	84.5 ± 10.4 (74.5–96.7)	N/A
Rest time between games (s)	134.2 ± 9.5 (128.7–145.2)	147.1 ± 22.9 (130.9–163.3)	138.6 ± 7.8 (132.9–147.4)	143.7 ± 7.4 (135.1–148.3)	N/A
Rest time between rallies (s)	22.9 ± 10.6 (8.0–45.6)	17.2 ± 4.4 (8.8–22.1)	24.9 ± 3.4 (21.4–30.0)	22.8 ± 4.0 (18.7–28.6)	N/A
Work density (work/rest)	0.4 ± 0.1 (0.3–0.5)	0.4 ± 0.1 (0.3–0.5)	0.2 ± 0.0 (0.2–0.3)	0.4 ± 0.0 (0.3–0.4)	N/A
External loads	Men's singles	Women's singles	Men's doubles	Women's doubles	Mixed doubles
Distance covered (m/min)	44.6 ± 1.6 (42.6–46.2)	43.5 ± 0.9 (42.8–44.5)	N/A	N/A	N/A
Jumps (N/min)	0.9 ± 0.0 (0.9–0.9)	0.8 ± 0.1 (0.7–0.9)	N/A	N/A	N/A
Accelerations (N/min)	25.9 ± 0.6 (25.3–26.4)	24.7 ± 0.2 (24.4–25.0)	N/A	N/A	N/A
Peak speed (km/h)	10.9 ± 0.1 (10.7–11.0)	10.7 ± 0.3 (10.4–11.0)	N/A	N/A	N/A
Internal loads	Men's singles	Women's singles	Men's doubles	Women's doubles	Mixed doubles
Peak heart rate (bpm)	192.3 ± 2.5 (188.7–194.5)	189.2 ± 12.5 (180.3–198.0)	N/A	N/A	N/A
Average heart rate (bpm)	162.6 ± 9.9 (138.3–175.7)	168.6 ± 10.5 (141.0–175.0)	N/A	N/A	N/A
Energy expenditure (kJ/min)	57.4 ± 15.1 (46.7–68.0)	45.4 ± 0.7 (44.9–45.9)	N/A	N/A	N/A
Blood lactate (mmol/L)	5.4 ± 3.1 (1.9–10.1)	2.2 ± 0.4 (1.9–2.5)	N/A	N/A	N/A
Fluid intake (L/h)	0.9 ± 0.2 (0.7–1.1)	0.9 ± 0.2 (0.7–1.0)	N/A	N/A	N/A
Sweat rate (L/h)	1.1 ± 0.1 (1.0–1.1)	1.0 ± 0.0 (1.0–1.0)	N/A	N/A	N/A
pH (level)	6.5 ± 0.2 (6.3–6.7)	6.5 ± 0.2 (6.3–6.7)	N/A	N/A	N/A
Dehydration level (%)	0.3 ± 0.1 (0.2–0.5)	0.3 ± 0.1 (0.2–0.5)	N/A	N/A	N/A

Minimum two studies or one study with minimum two data groups were used to calculate the mean.

BPM, beats per minutes; kJ/min, kilojoule per minute; km/h, kilometre per hour; L/h, liters per hour; m/min, meter per minute; mmol/L, millimole per liter; N/A, not available; N/min, newton per minute; n, number; n/s, number per seconds; pH, potential of hydrogen; s, seconds.

#### 3.4.1 Differences between singles category in both sexes

**Figure 2:**
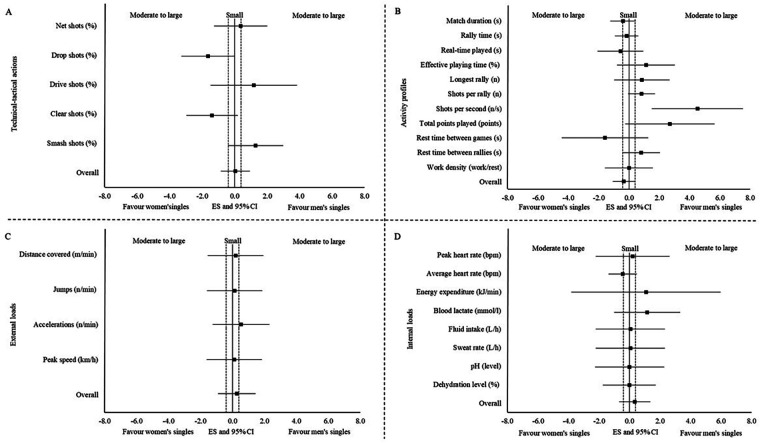
Individual and overall ESs and associated 95% confidence intervals with respect to differences between singles category in both sexes regarding **(A)** technical-tactical actions, **(B)** activity profiles, and **(C)** external and **(D)** internal loads. The dashed vertical lines present thresholds for small effect sizes; solid lines present zero effect sizes. BPM, beats per minutes; kJ/min, kilojoule per minute; km/h, kilometre per hour; L/h, liters per hour; m/min, meter per minute; mmol/L, millimole per liter; n/min, newton per minute; n, number; n/s, number per seconds; pH, potential of hydrogen; s, seconds.

Figure 2 shows the individual and overall ESs regarding differences in match-play outcome measures between men's and women's singles categories. Concerning technical-tactical actions, men performed largely more drive (9.9 ± 11.9 vs. 4.6 ± 3.7%; ES = 1.18 ± 0.96) and smash shots (20.3 ± 9.4 vs. 14.6 ± 6.3%; ES = 1.29 ± 0.79) than women. All further individual differences were small (ES = 0.37). The overall ES for technical-tactical actions was small (ES = 0.04 ± 0.43), favouring men's singles. Regarding activity profiles, men had a largely higher effective playing time (34.1 ± 5.3 vs. 29.0 ± 6.6%; ES = 1.13 ± 0.78), longest rally (41.7 ± 1.7 vs. 32.2 ± 4.1 n; ES = 0.86 ± 0.75), shots per rally (8.9 ± 2.1 vs. 6.9 ± 1.1 n; ES = 0.82 ± 0.43), shots per second (1.0 ± 0.2 vs. 0.8 ± 0.2 shots/s; ES = 4.52 ± 1.30), total points played (77.0 ± 7.5 vs. 42.3 ± 20.6 points; ES = 2.71 ± 1.15), and rest time between rallies (22.9 ± 10.6 vs. 17.2 ± 4.4 s; ES = 0.81 ± 0.56) than women. All further differences were small (ES = 0.00). The overall ES for the activity profiles was small (ES = −0.34 ± 0.37), favouring women's singles. No large differences were observed for external loads between men's and women's single categories. However, men executed moderately more acceleration (25.9 ± 0.6 vs. 24.7 ± 0.2 n/min; ES = 0.54 ± 0.72) than women. All further differences were small (ES = 0.11–0.20). The overall ES for the external loads was small (ES = 0.27 ± 0.56), favouring men's singles. Regarding internal loads, men had a largely higher energy expenditure (57.4 ± 15.1 vs. 45.4 ± 0.7 kJ/min; ES = 1.08 ± 1.14) and blood lactate (5.4 ± 3.1 vs. 2.2 ± 0.4 mmol/L; ES = 1.16 ± 0.88) than women. All further differences were small (ES = 0.00–0.20). The overall ES for internal loads was small (ES = 0.34 ± 0.50), favouring men's singles.

#### 3.4.2 Differences between the doubles category in both sexes and mixed

**Figure 3:**
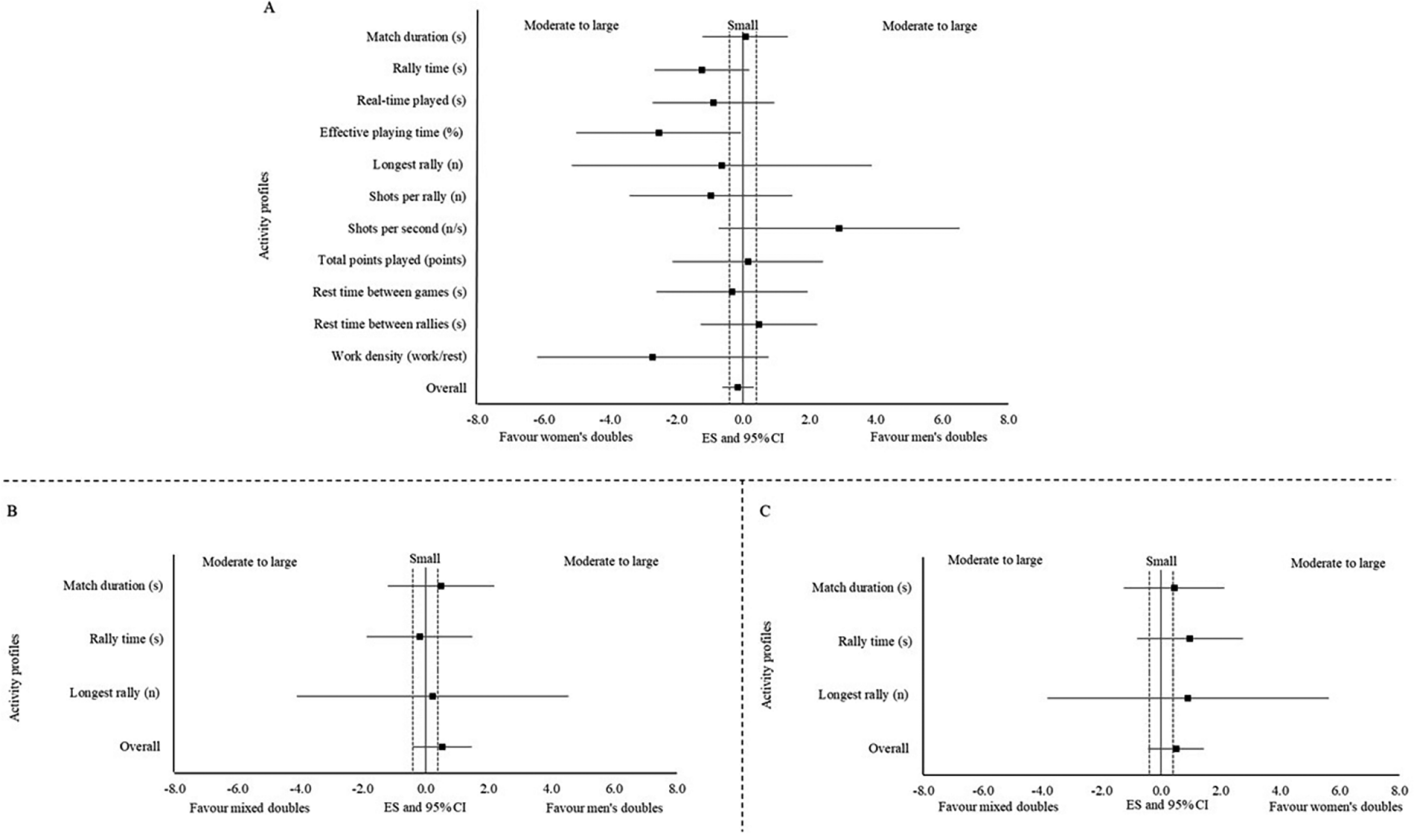
Individual and overall ESs and associated 95% confidence intervals with respect to differences between the doubles category in **(A)** both sexes, **(B)** men's and mixed, and **(C)** women's and mixed regarding the activity profiles. The dashed vertical lines present thresholds for small effect sizes; solid lines present zero effect sizes. n, number; n/s, number per seconds; s, seconds.

Figure 3 shows the individual and overall ESs for differences in doubles categories between sexes and mixed activity profiles. Regarding activity profiles between men's and women's doubles categories, men's doubles categories performed largely more shots per second (1.5 ± 0.0 vs. 1.3 ± 0.1 n/s; ES = 2.90 ± 1.31) than women's doubles. Furthermore, men's doubles had moderately higher rest time between rallies (24.9 ± 3.4 vs. 22.8 ± 4.0 s; ES = 0.48 ± 0.72) than women's doubles. All further differences were small (ES = 0.07–0.15). The overall ES for the activity profiles was small (ES = −0.15 ± 0.24), favouring women's doubles. Regarding activity profiles between men's and mixed doubles categories, men's doubles had a moderately higher match duration (3,106.2 ± 665.0 vs. 2,653.0 ± 176.5 s; ES = 0.49 ± 0.72) than mixed doubles. All further differences were small (ES = 0.23). The overall ES for the activity profiles was moderate (ES = 0.54 ± 0.45), favouring men's doubles. Regarding activity profiles between women's and mixed doubles categories, women's doubles had a largely higher rally time (9.7 ± 0.9 vs. 6.8 ± 1.1 s; ES = 0.97 ± 0.75), and longest rally (57.4 ± 1.4 vs. 39.0 ± 3.1 n; ES = 0.90 ± 1.10) than the mixed doubles. Furthermore, women's doubles had moderately higher match duration (3,040.1 ± 743.0 vs. 2,653.0 ± 176.5 s; ES = 0.44 ± 0.72), than the mixed doubles. The overall ES for activity profiles was moderate (ES = 0.50 ± 0.45), favouring women's doubles.

#### 3.4.3 Differences between singles and doubles categories in both sexes

**Figure 4:**
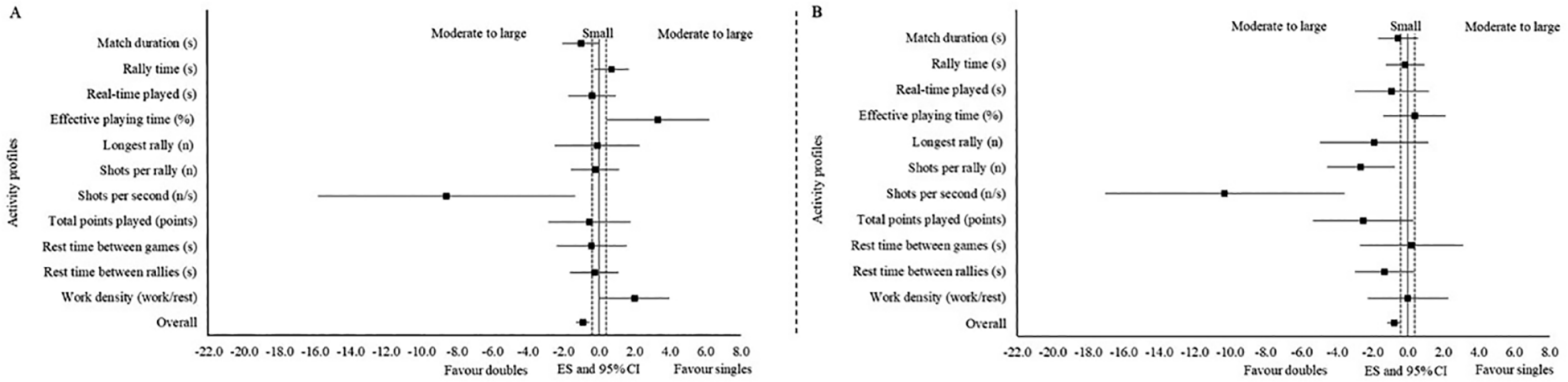
Individual and overall ESs and associated 95% confidence intervals with respect to differences between singles and doubles categories in **(A)** men and **(B)** women regarding the activity profiles. The dashed vertical lines present thresholds for small effect sizes; solid lines present zero effect sizes. n, number; n/s, number per seconds; s, seconds.

Figure 4 shows the individual and overall ESs with respect to differences in the single- and double-categories between sexes regarding activity profiles. In men's activity profiles, singles categories had a largely higher effective playing time (34.1 ± 5.3 vs. 19.2 ± 2.3%; ES = 3.32 ± 1.19) and work density (0.4 ± 0.1 vs. 0.2 ± 0.0 work/rest; ES = 2.00 ± 0.85) than doubles categories. Furthermore, singles categories had moderately higher rally time (8.8 ± 2.0 vs. 6.5 ± 0.7 s; ES = 0.69 ± 0.48) than doubles categories. The overall ES for activity profiles was large (ES = −0.90 ± 0.20), favouring doubles categories. No large differences were observed in women's activity profiles. All further differences were small (ES = 0.00–0.38). The overall ES for activity profiles was large (ES = −0.76 ± 0.21), favouring doubles categories.

## 4 Discussion

This systematic review is the first to investigate differences in match-play data according to the five playing categories in badminton. The main finding was that each playing category places specific demands on the players, which are important to consider when optimising training and testing procedures.

No previous systematic review of match-play data in badminton directly supports our findings. The only previous systematic review of badminton focused on health outcomes (19). Additionally, two other previous reviews focused on a similar topic to ours, but one of them was conducted narratively (1) and the other solely focused on internal loads across several racquet sports (20). In our review, we discovered that most studies investigated men and world-class players during official matches. Compared with other playing categories, men's singles were investigated most often. Moreover, most studies focused on activity profiles as an outcome measure. Therefore, research on women, recreationally active players, doubles categories, and external load measures is lacking. This research gap should be addressed by future studies. With this in mind, advanced methodological approaches may be helpful to investigate match-play data more profoundly in each of the five playing categories during the next years. For example, conducting cross-sectional and longitudinal studies on women, recreationally active players, and doubles categories by utilizing wearable technology such as local positioning systems (67) for external, and also extrapolated internal (metabolic) load measures (68) may contribute to close the here observed research gab.

In the men's singles category, the overall ESs for match-play data regarding technical-tactical actions, activity profiles, and external and internal loads showed only small differences (ES = −0.34 to 0.34) compared with women's singles (Figures 2A–D). However, some individual ESs showed up to large differences (ES ≤ 4.52), favouring the men's single category. For instance, men performed moderately to largely more drive shots, smash shots, accelerations, effective playing time, longest rallies, shots per rally, shots per second, total points played, and rest time between rallies than women. Additionally, men had a largely higher energy expenditures and blood lactate than women (Figure 2D). These observations are supported by a previous review stating that men's singles exhibit more aggressive attacking characteristics and are more physically demanding than women's singles (1, 2, 41–43, 48, 52). Thus, men's singles often require longer rest times between rallies (1). Differences in particularly muscular performance between men and women can explain these findings (69). A larger muscle mass allows men to engage in more aggressive play than women (69). Consequently, men's singles players need longer recovery time to maintain high energy levels for continued aggressive attacks (1, 70). Overall, these outcomes suggest that men's singles matches are characterised by more aggressive attacks at higher intensities during rallies, interspersed with longer recovery times than women's singles.

Regarding sex differences in the singles categories, some individual ESs also showed up to large differences (ES ≤ −1.63), favouring the women's single category (Figure 2A). For instance, women performed moderately to largely more drop shots, clear shots, real-time play, rest time between games and had a higher average heart rate than men (Figures 2B, D). These results are supported by previous studies stating that the women's singles category tends to be more defensive with a dominance of smoother shots than men's singles, resulting in longer real-time play (1, 2, 41, 43, 51, 52). In contrast, Valldecabres et al. (48) showed that the women's singles category was dominant in executing powerful shots (such as drive shots) compared with the men's singles category. However, this study (48) was based only on final matches, whereas other studies focused on matches across all phases (43, 51, 52). Sex differences in muscle masses (71) and hormones (69, 72) as well as cardiovascular (73) and neuro-muscular characteristics (74) including aerobic and anaerobic capacities (75) may contribute to the variations observed the in activity profiles between women and men. For example, women's singles players, characterized by lesser muscle mass, engage in less aggressive play with longer rallies (69). Consequently, defensive characteristics are more evident in women's singles (1, 2). Overall, the outcomes suggest that the women's singles category is characterised by a more defensive play with smoother shoots, leading to longer real-time plays than in men's singles.

In the doubles category, the overall ESs for the activity profiles were small to moderate (ES = −0.15, 0.50), favouring women's doubles over men's and mixed doubles, respectively (Figures 3A, C). Specifically, some individual ESs showed up to large differences (ES ≤ −2.72), favouring the women's doubles category (Figure 3A). The overall ES for activity profiles was moderate (ES = 0.54), favouring men's doubles over mixed doubles (Figure 3B). In contrast, the overall ESs for activity profiles were large (ES = −0.76 to −0.90), favouring the doubles category for both sexes (Figures 4A, B). These results are supported by previous studies (4, 43, 51, 53). For example, Gawin et al. (43) stated that the work density of women (30.1%) was greater than that of men and mixed doubles (approximately 20% for each). This statement supports our findings, where our results (ES = −2.72) showed that women's doubles had a largely higher work density than men's doubles (Figure 3A). No comparison of work density between women and mixed doubles was observed; however, rally time (ES = 0.97) was largely higher in women's doubles than in mixed doubles. A previous study assumed that shortening coverage areas could intensify match play by increasing shot frequency (4). This statement is supported by our findings, where shots per rally and shots per seconds showed up to large differences (ES ≤ −10.31), favouring the doubles category for both sexes (Figure 4B). These results suggest that the shortening coverage area prompts double players to hit the shuttlecocks earlier, thereby increasing shot frequency. Overall, the outcomes suggest that women's doubles categories have greater work density and rally time than men's and mixed doubles categories. Additionally, the results of activity profiles showed an increase in shot frequency for the doubles compared to the singles category, but no clear conclusion concerning the playing demands in doubles are possible due to a lack of external and internal load measures on an individual player level yet.

Overall, these findings support that badminton shares some demands in common with further racquet sports as tennis and squash (76). However, our subgroup analysis indicates that each category in badminton places specific demands on players. From a practical perspective, our results can serve as a framework to design training, testing and talent identification procedures in badminton. As a general implementation, we carefully recommend such practical aspects based on our main findings summarized in Table 7. Specifically, these recommendations were derived from our match-play data outcomes measure of each category. Therefore, suggested energy contributions serve as foundation for guiding training and testing procedures, which should be worked out by badminton experts. However, our general recommendations should be treated with caution due to the large 95% confidence intervals of some ESs (e.g., longest rally, shots per second, and energy expenditure), indicating a large heterogeneity of the underlying data, which limits a generalization. Therefore, more research is needed not only to prove the observed heterogeneity, but also to evaluate the provided general recommendations, as we have not investigated them here.

**Table 7:** General practical recommendations for training and testing procedures for men's and women's singles according to main findings of our study.

Categories	Main findings of match-play outcome measures	Suggested energy contributions	Recommended training procedures	Recommended testing procedures
Men's singles	• More drive and smash shots • More shots per second and total played points as well as longer rest time between rallies • More accelerations • Higher energy expenditure and lactate concentration	• Aerobic↑ • Anaerobic (alactic and lactic)↑↑	• High-intensity interval training or drill shots with shorter work and longer recovery • High-load resistance training with few repetitions focused on muscular power and additional plyometric training	• Ramp-like running protocol to determine maximum oxygen uptake and ventilatory thresholds • Repeated non-linear sprint ability protocols with shorter work and longer recovery to determine maximum and mean sprint times • Muscular power protocols to determine rate of force development
Women's singles	• More drop and clear shots • Longer real-time played • Higher heart rate	• Aerobic↑↑ • Anaerobic (alactic and lactic)↑	• Moderate-intensity interval training or drill shots with longer work and shorter recovery • Moderate-load resistance training with numerous repetitions focused on muscular endurance and additional plyometric training	• Ramp-like running protocol to determine maximum oxygen uptake and ventilatory thresholds • Repeated non-linear sprint ability protocols with longer work and shorter recovery to determine maximum and mean sprint times • Muscular endurance protocols to determine time to failure

General practical recommendation for the doubles categories could not be provided due to lack of available match-play data outcomes.

↑– Relative amount of energy contribution.

This systematic review had several limitations. First, a meta-analysis could not be conducted due to the large heterogeneity of the included studies and their data. However, ESs were calculated as an established alternative statistical approach (27, 28). Second, we did not investigate differences in playing levels between players, because this would have significantly exceeded the scope of our review. Therefore, further studies are needed to address these issues.

## 5 Conclusion

There are differences in match-play data according to the five playing categories in badminton, each category placing specific demands on the players. Men's singles are characterised by explosive movements at high intensity, indicating that not only a high aerobic, but also a sufficient amount of anaerobic capacity is needed. In contrast, women's singles are characterised by more defensive play and smoother shoots, suggesting a greater aerobic demand compared to men's singles. In the doubles category, the frequency of shots is increased, but no clear conclusion concerning the playing demands are possible due to a lack of outcome measures on an individual player level. Nevertheless, specific training and testing procedures are essential for the players to prepare them according to the specific demands of each category, what should be considered by scientists and coaches.

## Data Availability

The raw data supporting the conclusions of this article will be made available by the authors, without undue reservation.
